# Early Life Nutrition Factors and Risk of Acute Leukemia in Children: Systematic Review and Meta-Analysis

**DOI:** 10.3390/nu15173775

**Published:** 2023-08-29

**Authors:** Ambroise Kouame Kintossou, Jessica Blanco-Lopez, Isabel Iguacel, Silvia Pisanu, Claudia Choma Bettega Almeida, Eva Steliarova-Foucher, Ciska Sierens, Marc J. Gunter, Elena J. Ladas, Ronald D. Barr, Koen Van Herck, Zisis Kozlakidis, Inge Huybrechts

**Affiliations:** 1 International Agency for Research on Cancer, 69007 Lyon, France; kkintossou@gmail.com (A.K.K.); steliarovae@iarc.who.int (E.S.-F.); kozlakidisz@iarc.who.int (Z.K.); huybrechtsi@iarc.who.int (I.H.); 2 Biobank, Pasteur Institute of Côte d’Ivoire, Abidjan 01 BP 490, Côte d’Ivoire; 3 Faculty of Health Sciences, University of Zaragoza, 50009 Zaragoza, Spain; iguacel@unizar.es; 4 Section of Microbiology and Virology, Department of Biomedical Sciences, University of Cagliari, 09124 Cagliari, Italy; s.pisanu6@gmail.com; 5 Department of Nutrition, Federal University of Paraná, Curitiba 80060-000, Brazil; clauchoma@gmail.com; 6 Department of Public Health and Primary Care, Ghent University, 9000 Ghent, Belgium; ciskasierens@hotmail.com (C.S.); koen.vanherck@kankerregister.org (K.V.H.); 7 Faculty of Medicine, School of Public Health, Imperial College London, London SW7 2AZ, UK; m.gunter@imperial.ac.uk; 8 Division of Hematology, Oncology and Stem Cell Transplantation, Department of Pediatrics, Columbia University Irving Medical Center, New York, NY 10032, USA; ejd14@cumc.columbia.edu; 9 Departments of Pediatrics, Pathology and Medicine, McMaster University, Hamilton, ON L8S 4L7, Canada; rbarr@mcmaster.ca

**Keywords:** childhood acute leukemia, early child’s diet, vitamin K administration, breastfeeding

## Abstract

Acute leukemia commonly occurs in young children with peak incidence at the age of 2–5 years. However, the etiology is still unclear and many preventable risk factors still deserve to be reviewed. The focus of this systematic review and meta-analysis is to summarize the evidence concerning early life nourishment (breastfeeding, early life diet), neonatal vitamin K administration and the risk of acute leukemia. All epidemiological studies published up to June 2023 and assessing diet-related risk factors for childhood acute leukemia were identified in two electronic databases (PubMed and Web of Science), with no limits on publication year or language. A total of 38 studies (37 case–control studies and 1 study with pooled analysis) were included. The published risk estimates were combined into a meta-analysis using the Generic Inverse Variance method. The current evidence shows that breastfeeding (yes vs. no) has a protective effect against acute lymphoblastic leukemia (odds ratio = 0.85; 95% CI, 0.76–0.94). Evidence related to the role of other studied factors (foods and supplements) is inconclusive. Further research into the potential role of diet in early life and the risk of acute leukemia is needed to develop prevention strategies at population level. Review Registration: PROSPERO registration no. CRD42019128937.

## 1. Introduction

Childhood cancer is a notable cause of morbidity and mortality, with incidence rising from 124 to 140 per million person years between the eighties and 2000s in populations covered by cancer registration [1]. Acute leukemia (AL) is the most common type of cancer in children under 15 years old in most populations. The reported world age-standardized incidence rate of leukemia is 46.4 per million per year in children aged 0–14 years, and 28.5 per million in adolescents aged 15–19 years [1]. As AL is a rare disease, retrospective case–control design was used to study potential risk factors. Case control studies are known to be subjected to bias in exposure assessment [2,3]. The methodological limitations of retrospective case–control studies can partially explain the inconsistent results of the association between diverse exposures and leukemia etiology. Thus, apart from exposure to ionizing radiation, specific types of chemicals (e.g., benzene, chemotherapy), and certain genetic variations and syndromes, our understanding of the causes of AL in children is limited [2,4,5].

Almost 80% of AL cases are lymphoblastic lineage (ALL) and 15% are myeloid lineage (AML) in children from 0 to 14 years old [6], although incidences may vary by diverse factors (e.g., region, ethnicity) [7]. Age standardized rates of leukemia varies from 12.5 (Sub-Saharan Africa) to 65.4 (white Hispanics in USA) per million person years [1]. It is noteworthy that the differential incidence across geographic regions may be due to underdiagnosis in low-income settings [8]. In addition, considering a higher incidence around two to five years of age, it is reasonable to hypothesize that exposures (lifestyle and environmental exposures) during the perinatal and early infancy period could contribute in the pathogenesis of childhood leukemia [1,2,9]. Diet and supplementation provide nutrients for our daily life, but they are also implied in complex pathways (e.g., cellular replication, hormone regulation, immune response) that may modulate the hazard of developing any cancer, including leukemia [2,9].

Therefore, in this systematic review, we focused on the likely role of breastfeeding, early child’s diet, and neonatal administration of vitamin K on the risk of AL in children. The combined evidence could be used to assess the development of preventive strategies on a population level to reduce the incidence and mortality of leukemia.

## 2. Materials and Methods

### 2.1. Definition of the Outcome

For the systematic review, the incidence of AL in children aged 0–14 years and adolescents aged 15–19 years was the main outcome. Acute lymphoblastic leukemia (ALL) and acute myeloid leukemia (AML) were included as subtypes of leukemia [6,10,11].

### 2.2. Definition of the Risk Factors

The considered risk factors were breastfeeding (breastfed, lactation, infant feeding), early child’s diet circa first 3 years of life (diet, food intake, nutrition, supplement), and the neonatal administration of vitamin K.

### 2.3. Systematic Review Registration

PROSPERO was used to record the protocol details of this systematic review on registration number CRD42019128937.

### 2.4. Search Strategy

**Figure 1:**
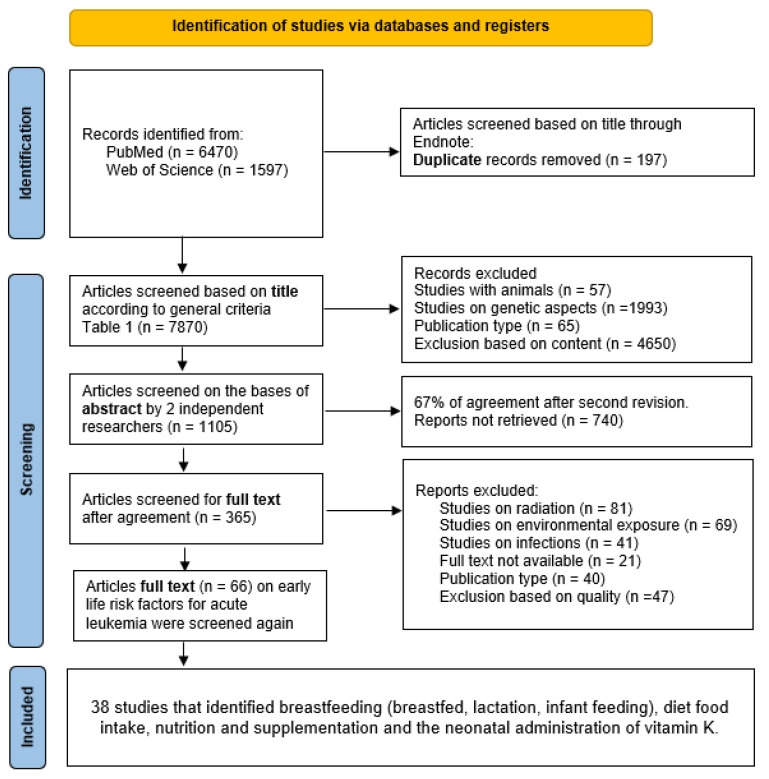
Final search strategy.

To identify early life nutritional factors that have been studied in relation to leukemia risk, we conducted a comprehensive multi-level literature search using Medline (via PubMed) and Web of Science. The first level of analysis for this systematic review consisted of an exploratory search for studies on leukemia in infants, children and adolescents and early life exposures (pregnancy to early life—first three years). This first search was conducted in December 2017, without being limited by language or publication date. A total of 66 studies were identified in this search; at this stage, exposures during pregnancy and early life were included. Based on these results, and after sub-grouping the exposures, we were able to further restrict the keywords and carry out a more targeted search, updated in June 2023, which focused on the nutritional exposures identified in early life, described in Supplementary Table S1. For all concepts, database-specific thesauri were used to identify relevant synonyms. The final search strategy is illustrated in Figure 1.

### 2.5. Selection of the Studies and Quality Assessment

Studies were included if they provided estimates of effect (relative risk (RR), odds ratio (OR) or hazard ratio (HR) and 95% confidence intervals (CI)), or if they provided sufficient data to calculate estimates for different exposure levels.

Table 1 lists the inclusion and exclusion criteria used in the review. PRISMA guidelines were followed [12] (Supplementary Table S2). All references found through the search questions (Figure 1) were imported into Endnote software (Version x8, Clarivate Analytics, Philadelphia, PA, USA). Two independent authors (AK-K and JB-L) searched for publications in each database (PubMed and Web of Science). Any uncertainty regarding the selection of a given article was resolved by a third reviewer (IH) and discussed within the working group, if necessary. The PICOS criteria for included studies are listed in Table 2, and the selected studies included for each extracted factor are summarized in Table 3. Twenty-one articles were excluded because the full text was not available; it was not possible to obtain a copy, even when requesting the authors.

Quality control of the selected articles was then carried out using the checklist established by Fowkes et al. to ensure the inclusion of high-quality studies [13]. This checklist includes questions on study design and sample, characteristics of the control group, quality of measurements and results, completeness, and influence of bias. A major flaw identified for a given criterion is indicated by a ++ sign. The + sign has been applied to a criterion with a minor defect. In the absence of a defect, the sign 0 (nil) was applied. If a study obtained a score of ++ for more than one criterion in this checklist, it was excluded. The results of the quality control are shown in Supplementary Table S3, and details of the excluded studies are shown in Supplementary Table S4.

**Table 1:** Inclusion and exclusion criteria.

Inclusion and Exclusion Criteria
General inclusion criteria
All studies describing early life nutritional exposures and its influence on the incidence leukemia in children and adolescents were included in the systematic review. Pooled analyses including new data were also included in the review.
General exclusion criteria
Studies on animals or on the genetic aspects Full text not available Studies without original data (e.g., editorial, review, reports, guidelines)
Exclusion criteria based on content
Studies of leukemia in children who already have other concomitant disorders (e.g., Down syndrome, congenital malformations) Studies of the association between radiation and leukemia (e.g., X-rays during pregnancy; living near a nuclear power station) Studies of the association between environmental exposure and leukemia (e.g., air pollution, exhaust fumes, pesticides) Studies on the association between infectious diseases and leukemia (maternal infections during pregnancy, illness of the child in the early years of life, presence in the nursery)
Exclusion criteria based on study scope and data quality
Studies with less than 50 cases Insufficient quality based on the checklist of Fowkes et al.

**Table 2:** PICOS criteria for inclusion and exclusion of studies.

Parameter	Criterion
Participants	Children and adolescents diagnosed with acute leukemia
Interventions	Early life nourishment (including breastfeeding and early child’s diet) and vitamin K administration
Control/comparator group	Healthy children and adolescents
Outcomes	Childhood acute leukemia (AL, ALL, AML)
Study design	Observational studies with a comparison group (cohort studies, case–control studies)

**Table 3:** Overview of the included studies.

Extracted Factors (Early Life Nutrition Factors)	Number of Included Articles ^1^	References
Breastfeeding	25	[14,15,16,17,18,19,20,21,22,23,24,25,26,27,28,29,30,31,32,33,34,35,36,37,38]
Early child’s diet	6	[16,23,39,40,41,42]
Vitamin K administration	9	[43,44,45,46,47,48,49,50,51]

^1^ A total of 37 case–control studies and one study with pooled analysis were included.

### 2.6. Data Extraction

Table 4 summarizes the included studies according to name, design, sample size, age at diagnosis and country of study population. Key data extracted from all included studies, effect estimates (RR, OR or HR, 95% CI) and confounding variables tested are presented in Supplementary Tables S5–S7.

### 2.7. Quantitative Meta-Analysis

A meta-analysis was performed to estimate which factors had an impact on AL, ALL or AML. For each factor included, the choice to perform an additional quantitative analysis was based on the number (at least two studies) and homogeneity of the included studies (e.g., studies limited to infants or univariate analyses without adjustments were not pooled), and data from overlapping studies were also excluded. We used the reported adjusted odds ratio (OR) in case–control studies and the relative risk (RR) in cohort studies to calculate the overall effect. Analyses were performed using the generic inverse variance method, in which the weight of each study is equivalent to the inverse of the variance of the effect estimate [52]. A random-effects model was used where there was evidence of heterogeneity. For exposures reported at different levels, we pooled an estimate for a binary exposure status (exposed vs. unexposed). Heterogeneity was assessed using the I2 statistic, where 0% indicates perfect homogeneity and 100% complete heterogeneity [53]. Data were analyzed using Review Manager (RevMan) V.5.4.1, Cochrane, London, UK [54]. The results are summarized in Table 5.

## 3. Results

### 3.1. Selected Studies

The results of the search strategy and the process of selecting studies for review are illustrated in Figure 1. The preliminary search yielded 7870 studies, which were selected based on their titles. After evaluation of titles and abstracts, 365 articles met the criteria for full-text review. Consequent review of the full-text reports of the 66 selected publications identified 38 eligible studies (37 case–control studies and one study with pooled analysis).

### 3.2. Characteristics of the Included Studies

Table 3 shows the number of studies included in each extracted exposure. The detailed characteristics (author, study period and location, year of publication, number and age of participants, study design, control source and extracted exposure variables) of each included study are presented in Table 4.

### 3.3. Quality Assessment

The quality assessment [13] of the included studies is presented in Supplementary Table S3. A common weakness detected in some studies was the lack of adjustment for possible confounding factors such as parental smoking status, maternal alcohol consumption and perinatal factors (e.g., birth weight, type of delivery). Supplementary Table S4 contains a list of the excluded studies. The confounding factors used in the analysis for each included study are presented in Supplementary Tables S5–S7.

### 3.4. Expousures and Detected Association with the Outcomes

#### 3.4.1. Breastfeeding

Twenty-five case–control studies studied the link between breastfeeding and risk of AL in children [14,15,16,17,18,19,20,21,22,23,24,25,26,27,28,29,30,31,32,33,34,35,36,37,38]. Results are reported in Supplementary Table S5.

From eleven case–control studies [14,16,19,22,24,28,30,31,33,35,37] that studied breastfeeding and AL, only five [16,19,22,31,37] reported a statistically significant protective effect of breastfeeding (most of them with never breastfed as reference). An increased risk for AL among not breastfed children (compared to children breastfed for >13 months) was obtained in another study [22].

Eighteen case control studies evaluated the association of breastfeeding with ALL [15,17,18,20,21,23,24,25,26,27,29,31,32,34,35,36,37,38]; of them, only nine [15,17,18,23,25,31,34,36,37] reported a statistically significative protective effect (most of them when compared with never breastfed as reference). The strongest association was found for 7–12 months of breastfeeding duration [36].

Nine case control studies [15,21,24,27,31,32,35,36,37] appraised the association of breastfeeding with AML. In one of them, a breastfeeding period of 7–9 months found to reduce the risk of AML [36], and other study described an increased risk of AML in children breastfed less than 6 months [15].

**Figure 2:**
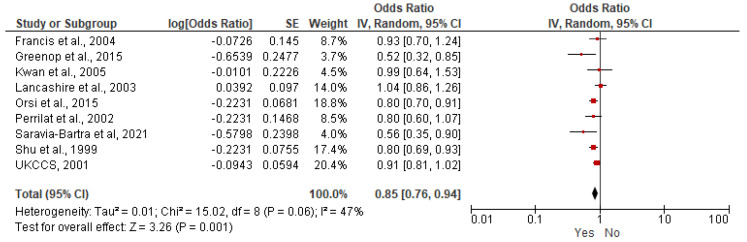
Random-effects model examining the association between breastfeeding (Yes versus No) and risk of childhood ALL [21,23,26,27,31,32,34,36,37]. Note: the individual estimate (OR) from the studies is represented by the red box, and the black diamond represent the estimate of the meta-analysis.

**Figure 3:**
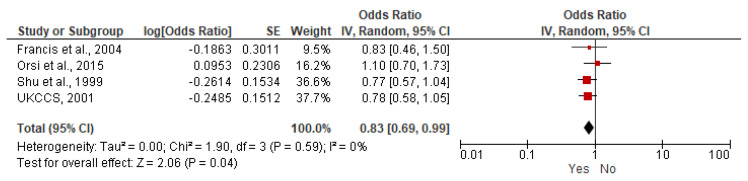
Random-effects model examining the association between breastfeeding (Yes versus No) and risk of childhood AML [21,31,36,37]. Note: the individual estimate (OR) from the studies is represented by the red box, and the black diamond represent the estimate of the meta-analysis. For each factor included, quantitative analysis was performed with at least two studies, results from studies limited to infants, univariate analysis without adjustment and overlapping data were excluded.

A meta-analysis of nine case–control studies comparing children breastfed (any duration) versus children not breastfed was performed (6400 children with ALL, 17,642 children without ALL). The age of participants ranged between less than 14 years old and less than 16 years old [21,23,26,27,31,32,34,36,37]. A decreased risk of ALL was found for breastfed children (OR = 0.85, 95% CI: 0.76–0.94, *I*^2^ = 47%) (Figure 2), and for AML (OR = 0.83, 95% CI: 0.69–0.99, *I*^2^ = 0%) (Figure 3. All the results are included in Table 5 and Supplementary Figures S1–S6.

#### 3.4.2. Early Life Diet

Six case control studies explored the association between children’s early diet and the risk of AL [16,23,39,40,41,42]. Results are reported in Supplementary Table S6.

Three case control studies [16,40,41] investigated the association between early diet (during the first 2 years of life) and the risk of childhood AL. One of them stated a lowered risk of AL with regular consumption of oranges/bananas and orange juice [40]. Another study reported a lower risk of AL with the frequent (weekly) consumption of bean-curd and vegetables, and the risk increased with frequent consumption of cured meat/fish [41]. Iron supplementation was shown beneficial in one [16]. This study also assessed the impact of early diet on a combined risk of developing AL and lymphoma, and found an increased risk when processed food was consumed (processed meats, salty snacks, and candy ≥ 3 times/week), and a reduced risk when diet included vegetables at least three times per week.

Among the three case control studies that investigated the association between food consumption and ALL [23,39,42], one reported an increased risk for added lipids (butter, margarine, seed and olive oils, and olives), and no significant association with consumption of fruits [39]. Another study found no association with food or supplements with the risk of ALL [23].

Our meta-analysis, performed on two studies [23,39], evaluated the association between the consumption of fruits and vegetables and the risk of ALL: none was found (Table 5 and Supplementary Figures S7 and S8).

#### 3.4.3. Neonatal Vitamin K Administration

Eight case–control studies [43,44,45,46,47,48,49,51] and one study with pooled analysis [50] investigated the association between neonatal intramuscular application of vitamin K and risk of AL in children (Supplementary Table S7).

The study with the pooled analysis [50] included some of the revised case control studies [43,46,47,48,49,51]. In two of these revised studies, vitamin K administration was found to increase the risk of AL [46] and ALL [48]. However, the pooled study did not find any statistically significative associations between the intramuscular administration of vitamin K and leukemia.

In a case–control study assessing intramuscular and oral administration of vitamin K, no association with the risk of ALL or AL was found in children aged one year or older [45]. Non-significant results were reported in another case–control study conducted in Scotland [44]. In contrast with these findings, intramuscular vitamin K in children younger than eight years of age was found to lower the risk of ALL, although this effect was no longer significant after adjustments [55].

Our meta-analysis, performed on two studies [45,50], evaluated the association between the neonatal administration of vitamin K and the risk of ALL: none was found (Table 5 and Supplementary Figure S9).

## 4. Discussion

With this systematic review and meta-analysis, we aimed to integrate the available scientific research on nutritional factors in early life (birth to age 3) and the impact of vitamin K administration that could be linked to the development of childhood acute leukemia.

Our systematic review and meta-analyses indicate a protective effect of breastfeeding against the risk of ALL (the minimum duration of breastfeeding in the included studies was less than 1 month or less than 3 months). All case–control studies that have investigated the impact of breastfeeding have found a decreased risk for breastfed children. In line with our findings, a meta-analysis of 33 studies published in 2021 compared systematic breastfeeding with ever-breastfed versus non- or occasional breastfeeding (OR = 0.77, 95% CI: 0.65–0.91) and longer breastfeeding with shorter breastfeeding versus shortest and longest duration (OR = 0.77, 95% CI: 0.63–0.94), showing a protective effect [56]. Similar results were obtained in a previous meta-analysis of 17 studies published in 2015, which showed that, compared with no breastfeeding or shorter breastfeeding, any breastfeeding for 6 months or more was associated with a 20% lower risk of childhood leukemia (OR = 0.80, 95% CI: 0.72–0.90). In the same study, the meta-analysis of 15 studies indicated that always having been breastfed versus never having been breastfed was associated with an 119% lower risk of childhood leukemia (OR = 0.91, 95% CI: 0.80–1.04) [57]. Another meta-analysis showed that breastfeeding for at least one month could protect against childhood cancer (OR = 0.75, 95% CI: 0.63–0.89) [58].

In our meta-analyses, breastfeeding of any duration had a protective effect against ALL (OR = 0.85; 95% CI: 0.76–0.94) and for AML (OR = 0.83; 95% CI: 0.69–0.99). This protective effect of breastfeeding on both ALL and AML risk can be explained by the complex composition of the human milk. Breastfeeding provides nutrients (including antioxidants, fatty acids), maternal antibodies and immunological components (cytokines, immunoglobulins, etc.) and many other components that contribute to the advancement of the immune system and microbiota of the infant [9,59]. Breastfeeding not only contributes to the well-being of the infant but also has benefits for the mother with a protective effect against breast cancer (OR = 0.71, 95% CI: 0.59–0.84) [60]. In a larger context, breastfeeding has many other benefits for child health [61]. Breastfeeding was evaluated in multiple settings (low and high-income countries), some of the studies are recent, and findings are consistent with the protective effect.

Regarding children’s early diet, only a few studies evaluated the potential role of the food and supplements in leukemogenesis. The results from our meta-analysis, and those from a previous one, were not conclusive [62]. As mentioned in a previous publication, evaluating dietary intake is challenging, and some of the studies reported their findings by specific items instead of food groups which difficult comparisons. Most of the publications are case–control studies, and with this, design risk factors are evaluated retrospectively, which might be affected by bias recall. Although, the potential link of the maternal diet during gravidity on the risk of ALL among their offspring was demonstrated [11].

Supplementing neonates with vitamin K is a common practice that was introduced to prevent the hemorrhagic disease of the newborn (HDN). This supplementation may be administrated as oral or intramuscular to all the newborns, or only to those at higher risk of HDN. During the nineties, many studies consider the potential association between the intramuscular administration of vitamin K and the risk of AL [43,44,46,47,48,49,51]. The evaluation of the effect of vitamin K in childhood AL is difficult since almost all newborns in western countries over the past 10 years received this prophylaxis at birth. Our review does not show convincing evidence on the link between neonatal intramuscular application of vitamin K and leukemia risk in children.

While studies of breastfeeding could partly rely on registered data (more reliable), all dietary information were collected through interviews and their integrity could not be evaluated. Therefore, the protective effect of breastfeeding is more convincing than the identified effects of diet.

We would like to highlight the need for studies that explore early life dietary factors and the risk of developing childhood AL in a comprehensive way (standardized questionnaires), including general information on the main food groups and supplements, but also other approaches that might be considered (e.g., degree of food processing). Micronutrient supplementation, including neonatal administration of vitamin K, needs to be evaluated in a more comprehensive way, including dose, dilutants, etc., to provide the best evidence on their potential role in developing leukemia. It should be noted that AL is a rare disease, therefore most of the results come from retrospective case–control studies often with a restricted number of cases. This is an important consideration when interpreting results. It is also important to mention the proposed mechanism for leukemogenesis, which involves early pre-leukemic genetic changes in utero, followed by triggers (environmental exposures, infections, etc.) in early life that induce secondary genetic changes and an immune response [9].

## 5. Conclusions

After reviewing the available evidence that evaluates early life nutritional factors and the risk of childhood acute leukemia, the most abundant and conclusive evidence points to the protective role of breastfeeding. However, the reviewed studies did not provide sufficient evidence on the role of early diet in the development of childhood acute leukemia. Although further research is needed to develop and test cost-effective, evidence-based prevention strategies to combat acute leukemia in children, breastfeeding can be recommended as a factor contributing to prevention of childhood leukemia.

## Figures and Tables

**Table 4:** Characteristics of the included studies that included early life nutrition factors and risk of acute leukemia in children.

Study/Reference	Location and Study Period	Leukemia Type	Age Range (Years)	Cases/Controls	Control Source	Exposure Variable (s)	Source(s)/Assessment Tool
Abudaowd et al., 2021 [14]	Saudi Arabia, 2008–2019	ALL, AML	1–19	74/74	Community controls paired by age and sex	Breastfeeding	Telephone interview
Altinkaynak et al., 2006 [15]	Turkey, 1990–2000	ALL, AML	1–16	87/146	Randomly selected controls paired by sex and age	Breastfeeding	In person standardized interview
Amitay et al., 2016 [16]	Israel, 2005–2013	ALL, AML	1–19	121/384	Community controls paired by age, sex, and spoken language.	Breastfeeding, child’s early diet and supplements	In person standardized interview
Ansell et al., 1996 [43]	Oxford, Cambridge, Reading, 1951–1969	ALL	0–14	109/264	Randomly selected from births registers (paired by sex, hospital, and year-month of birth)	Vitamin K administration	Health registries
Bener et al., 2001 [17]	United Arab Emirates, 1983–1997	ALL	1–15	69/69	Healthy children (attending for immunization, periodic follow-up) or with minor medical problems	Breastfeeding	Standardized telephone interviews
Bener et al., 2008 [18]	United Arab Emirates, 1983–2004	ALL	0–15	103/169	Control were paired by sex and age	Breastfeeding	Standardized telephone interviews
Bonaventure et al., 2012 (ESCALE ^a^) [19]	France, 2003–2004	Acute leukemia	0–14	764/1681	Randomly selected jointly with the cases from French households with a landline telephone	Breastfeeding	Standardized telephone interview
Davis et al., 1988 [20]	Colorado (USA), 1976–1983	ALL	0–14	52/207	Community controls paired on sex, age (within 3 years), and telephone exchange area.	Breastfeeding	Telephone interview
Diamantaras et al., 2013 [39]	Greece, 1999–2003	ALL	5–14	121/121	Community controls paired on age, sex, Hispanic ethnicity, and maternal race	Child’s early diet	Interviewed face-to-face
Ekelund et al., 1993 [44]	Sweden, 1973–1989	Acute leukemia	0–18	196/200	National and health registries	Vitamin K administration	Health registries
Fear et al., 2003 (UKCCS ^b^) [45]	United Kingdom, 1992–1996	ALL	0–14	1001/4487	Community controls paired on sex, year-month of birth, and region of residence at diagnosis	Vitamin K administration	In person interview and child’s medical records
Francis et al., 2014 (CCCLS ^c^) [21]	California (USA), 1995–2008	ALL, AML	0–14	731/1464	Community controls paired by age, sex, Hispanic ethnicity, and maternal race	Breastfeeding	Birth records and interview (either in person or over the phone)
Gao et al., 2018 [22]	Zhejiang (China), 2008–2017	Acute leukemia	0–14	958/785	Community controls	Breastfeeding	Medical records and telephone interview
Golding et al., 1992 [46]	Bristol, 1971–1991	Acute leukemia	0–14	195/558	National and health registries	Vitamin K administration	Health registries
Greenop et al., 2015 (Aus-ALL ^d^) [23]	Australia, 2003–2006	ALL	0–15	322/679	Controls were enrolled by RDD ^h^ and paired by age, sex, and residence	Breastfeeding and child’s early diet	Mothers completed a food frequency questionnaires (FFQs)
Hardell et al., 2001 [24]	Sweden, 1988–1991	ALL, AML	0–14	124/119	Community controls paired on sex, and age	Breastfeeding	Medical records
Infante-Rivard et al., 2000 [25]	Quebec, Canada 1989–1995	ALL	0–10	491/493	Community controls were paired by age (within 2 years), sex, and region of residence.	Breastfeeding	Face-to-face interview
Kwan et al., 2004 (NCCLS ^e^) [40]	Northern California (USA), 1995–2002	Acute leukemia	0–14	328/328	Community controls paired on age, sex, Hispanic status, and maternal race	Child’s early diet	Mailed caregiver questionnaire and in person interview
Kwan et al., 2005 (NCCLS ^e^) [26]	Northern California (USA), 1995–2002	Acute leukemia	0–14	311/400	Community controls paired on age, sex, Hispanic ethnicity, and maternal race	Breastfeeding	Data collected by self-administered and in-person questionnaires
Lancashire et al., 2003 (OSCC ^f^) [27]	England, Wales and Scotland, 1972–1981	ALL	0–15	948/8052	Community controls paired on sex and date of birth	Breastfeeding	In person interview
Lingappa et al., 2018 [28]	Bangalore (India), 2015	Acute leukemia	0–15	120/120	Siblings of the cases	Breastfeeding	In person interview with a standardized questionnaire
Liu et al., 2009 [41]	Taiwan, 1997–2005	Acute leukemia	2–20	145/270	Community controls paired by age and sex.	Child’s early diet	In person interview with a standardized questionnaire
MacArthur et al., 2008 [29]	Canada, 1990–1994	ALL	0–14	399/399	Community controls paired on sex, age and area	Breastfeeding	In person interview and child’s medical records
McKinney et al., 1998 [47]	Scotland, 1991–1994	ALL	0–14	129/247	Community controls paired on sex and date of birth	Vitamin K administration	Medical records
Mohammadi et al., 2018 [30]	Sistan and Baluchestan province (Iran), 2016–2017	Acute leukemia	0–17	120/240	Hospitalized controls paired on age (within 2 years) with non cancer diseases (also no heart, blood or immunologic related diseases)	Breastfeeding	Medical records
Orsi et al., 2015 (ESTELLE ^a^) [31]	France, 2010–2011	ALL, AML	0–14	736/1421	Population controls frequency-matched on age and sex	Breastfeeding	Standardized telephone interview.
Parker et al., 1998 [48]	England, 1960–1991	Acute leukemia	0–14	207/621	Community controls paired on date and hospital of birth	Vitamin K administration	Clinical records
Passmore et al., 1998 [49]	England, and Wales 1968–1985	ALL	0–14	597/597	Controls paired for sex, month of birth, and hospital were chosen from the health registries.	Vitamin K administration	Health registries
Perrilat et al., 2002 [32]	France, 1995–1999	ALL	0–15	219/288	Hospitalized controls (from orthopedic and emergency department) paired on age, sex, ethnicity, hospital, and its catchment area	Breastfeeding	Standardized in person interview.
Petridou et al., 1997 [33]	Greece, 1993–1994	ALL	0–14	136/300	Controls paired on age (within six months for <3 years and 12 months for older children) and sex, hospitalized in the same institution for minor conditions	Breastfeeding	Interviewer-administered questionnaire
Sarasua et al., 1993 [42]	USA (Colorado), 1973–1986	ALL	0–14	56/56	Community controls paired by age, sex, and telephone exchange area. By RDD ^h^.	Child’s early diet	In person interview
Saravia-Bartra et al., 2021 [34]	Peru, 2015	ALL	0–13	112/229	Hospitalized controls (emergency department) without cancer and without Down syndrome	Breastfeeding	Medical records
Shu et al., 1995 [35]	China, 1986–1991	Acute leukemia	0–14	159/159	Controls lived with their natural parents and had no history of cancer	Breastfeeding	Cancer registries and face-to-face interview
Shu et al., 1999 (CCG ^g^) [36]	USA, Canada, Australia, 1989–1993	ALL	0–14	1744/1879	Community controls paired on year of birth, race, residential location (telephone area code and exchange)	Breastfeeding	Telephone interview
U. K. Childhood Cancer Study Investigators, 2001 (UKCCS ^b^) [37]	England and Wales, 1992–1996 Scotland, 1991–1994	ALL, AML	0–14	1615/3230	Community controls paired on year-month of birth, sex and region of residence at diagnosis	Breastfeeding	face-to-face standardized interview.
Van Duijn et al., 1988 [38]	Netherlands, 1973–1979	ALL	0–14	492/480	Community controls paired by sex and age (within 3 months)	Breastfeeding	Health registries and mailed questionnaire
von Kries et al., 1996 [51]	Germany (Lower Saxony), 1988–1993	Acute leukemia	0–14	272/218	National and health registries, and questionnaires	Vitamin K administration	Health registries and mailed questionnaires
Pooled analysis studies							
Roman et al., 2002 [50]	Bristol, Mainz, Oxford, Scotland, and Newcastle, 1968–1992	ALL	0–14	935/6338	Community controls paired on date and hospital of birth	Vitamin K administration	Clinical records

^a^ ADELE, ELECTRE, ESCALE and ESTELLE studies are part of Etude Sur les Cancers et les Leucémies de l’Enfant, Study on Environmental and Genetic Risk Factors of Childhood Cancers and Leukemia, ADELE and ELECTRE, which studied leukemias from 1995 to 1998; ESCALE (2002–2003) and ESTELLE (2010–2011) studied cancer and leukemia. ^b^ UKCCS: United Kingdom Childhood Cancer Study. ^c^ CCCLS: Cross-Canada Childhood Leukemia Study. ^d^ Aus-ALL: Australian Study of Causes of Acute Lymphoblastic Leukemia in Children. ^e^ NCCLS: Northern California Childhood Leukemia Study. ^f^ OSCC: Oxford Survey of Childhood Cancers. ^g^ CCG: Children’s Cancer Group. ^h^ RDD: Random Digit Dialing.

**Table 5:** Summary Odds Ratios (ORs) and 95% Confidence Intervals (Cis) obtained in the meta-analysis of data from referenced published studies of association of childhood leukemia with early life nutrition factors.

	Leukemia Type	N. of Studies/References	OR (95% CI)	Heterogeneity *I*^2^, *p*
Breastfeeding				
	Acute leukemia	-	-	
	ALL			
	≤6 months	5 [15,18,20,36,38]	1.56 (0.96–2.55)	84%, 0.07
	<3 months	4 [23,25,29,32]	0.84 (0.54–1.31)	71%, 0.44
	<1 month	2 [27,37]	1.03 (0.88–1.19)	0%, 0.73
	Yes	8 [21,23,26,27,31,32,34,36,37]	0.85 (0.76–0.94)	47%, 0.001
	AML			
	≤6 months	2 [15,36]	2.13 (0.32–13.97)	81%, 0.43
	Yes	4 [21,31,36,37]	0.83 (0.69–0.99)	0%, 0.04
Food group				
Fruits	Acute leukemia	-	-	-
	ALL	2 [23,39]	1.05 (0.88–1.25)	0%, 0.56
	AML	-	-	-
Vegetables	Acute leukemia	-	-	-
	ALL	2 [23,39]	1.09 (0.90–1.32)	0%, 0.37
	AML	-	-	-
Supplements				
Vitamin K	Acute leukemia	-		
	ALL	2 [45,50]	0.96 (0.82–1.12)	26%, 0.59
	AML	-		

## Data Availability

No new data were created or analyzed in this study. Data sharing is not applicable to this article.

## Supplementary Materials

The following supporting information can be downloaded at:https://www.mdpi.com/article/10.3390/nu15173775/s1, Supplementary Table S1: Search strings used in literature searches; Supplementary Table S2: PRISMA 2020 checklist Early life nutrition factors and risk of Acute Leukemia in children; Supplementary Table S3: Quality assessment of the studies included in the systematic review evaluated by the checklist proposed by Fowkes et al. [10]. Supplementary Table S4: List of excluded articles, specifying the reason for exclusion. Supplementary Table S5: Association between breastfeeding and the risk of acute leukemia in children. Supplementary Table S6: Association between early life diet and the risk of acute leukemia in children. Supplementary Table S7: Association between neonatal vitamin K administration and the risk of acute leukemia in children. Supplementary Figure S1: Random-effects model examining the association between breastfeeding (≤6 months) and risk of childhood ALL. Supplementary Figure S2: Random-effects model examining the association between breastfeeding (≤6 months) and risk of childhood AML. Supplementary Figure S3: Random-effects model examining the association between breastfeeding with no vs. yes response and risk of childhood ALL. Supplementary Figure S4: Random-effects model examining the association between breastfeeding with no vs. yes response and risk of childhood AML. Supplementary Figure S5: Random-effects model examining the association between breastfeeding (<3 months) and risk of childhood ALL. Supplementary Figure S6: Random-effects model examining the association between breastfeeding (<1 month) and risk of childhood ALL. Supplementary Figure S7. Meta-analysis of the association between the foods (fruits) and acute lymphoblastic leukemia. Supplementary Figure S8. Meta-analysis of the association between the foods (vegetables) and acute lymphoblastic leukemia. Supplementary Figure S9. Meta-analysis between intramuscular vitamin K given in neonatal period and ALL.
