# Supplementary material for: Early Life Nutrition Factors and Risk of Acute Leukemia in Children: Systematic Review and Meta-Analysis
Source: Nutrients. 2023 Aug 29;15(17):3775. doi: 10.3390/nu15173775 (PMC10489830; doi:10.3390/nu15173775)
Supplement: Supplementary file 1 [file nutrients-15-03775-s001.zip › nutrients-2537082-supplementary.pdf]

Supplementary table 1. Search strings used in literature searches*

**PubMed:**	
#1	Leukemia/etiology [MeSH]
#2	Leukemia/pathology [MeSH]
#3	Child [MeSH]
#4	Adolescent [MeSH]
#5	Early child's diet [MeSH]
#6	Vitamin K administration [MeSH]
#7	Breastfeeding [MeSH]
#8	Review [Publication Type]
#9	(#1 or #2) and (#3 or #4) and (#5 or #6 or #7) not #9
**Web science:**	
#1	Leukemia
#2	Child
#3	Adolescent
#4	Early child's diet
#5	Vitamin K administration
#6	Breastfeeding
#7	#1 and (#2 or #3) and (#4 or #5 or #6)

*Search strings containing keywords and database-specific terms (MeSH) were decided after testing different searches using Leukemia, Early child's diet, Vitamin K administration, Breastfeeding, and several synonyms as main concepts.

PRISMA 2020 Checklist

Supplementary table 2. PRISMA 2020 checklist: Early life nutrition factors and risk of acute leukemia in children: systematic review and meta-analysis.

Section and Topic	Item #	Checklist item	Reported on page # (or table #)
**TITLE**			
Title	1	Identify the report as a systematic review.	1 (Title)
**ABSTRACT**			
Abstract	2	Provide a structured summary including, as applicable: background; objectives; data sources; study eligibility criteria, participants, and interventions; study appraisal and synthesis methods; results; limitations; conclusions and implications of key findings; systematic review registration number.	1 (Abstract)
**INTRODUCTION**			
Rationale	3	Describe the rationale for the review in the context of existing knowledge.	1-2
Objectives	4	Provide an explicit statement of the objective(s) or question(s) the review addresses.	1-2
**METHODS**			
Eligibility criteria	5	Specify the inclusion and exclusion criteria for the review and how studies were grouped for the syntheses.	4 (Table 1)
Information sources	6	Specify all databases, registers, websites, organisations, reference lists and other sources searched or consulted to identify studies. Specify the date when each source was last searched or consulted.	3-4,11
Search strategy	7	Present the full search strategies for all databases, registers and websites, including any filters and limits used.	3 (Figure 1)
Selection process	8	Specify the methods used to decide whether a study met the inclusion criteria of the review, including how many reviewers screened each record and each report retrieved, whether they worked independently, and if applicable, details of automation tools used in the process.	3-4,11
Data collection process	9	Specify the methods used to collect data from reports, including how many reviewers collected data from each report, whether they worked independently, any processes for obtaining or confirming data from study investigators, and if applicable, details of automation tools used in the process.	11
Data items	10a	List and define all outcomes for which data were sought. Specify whether all results that were compatible with each outcome domain in each study were sought (e.g. for all measures, time points, analyses), and if not, the methods used to decide which results to collect.	5-10
	10b	List and define all other variables for which data were sought (e.g. participant and intervention characteristics, funding sources). Describe any assumptions made about any missing or unclear information.	5-10 (Table 4)
Study risk of bias assessment	11	Specify the methods used to assess risk of bias in the included studies, including details of the tool(s) used, how many reviewers assessed each study and whether they worked independently, and if applicable, details of automation tools used in the process.	3 (Supp. Table 2)
Effect measures	12	Specify for each outcome the effect measure(s) (e.g. risk ratio, mean difference) used in the synthesis or presentation of results.	3
Synthesis methods	13a	Describe the processes used to decide which studies were eligible for each synthesis (e.g. tabulating the study intervention characteristics and comparing against the planned groups for each synthesis (item #5)).	3-11

Section and Topic	Item #	Checklist item	Reported on page # (or table #)
	13b	Describe any methods required to prepare the data for presentation or synthesis, such as handling of missing summary statistics, or data conversions.	3-11
	13c	Describe any methods used to tabulate or visually display results of individual studies and syntheses.	3-11
	13d	Describe any methods used to synthesize results and provide a rationale for the choice(s). If meta-analysis was performed, describe the model(s), method(s) to identify the presence and extent of statistical heterogeneity, and software package(s) used.	11
	13e	Describe any methods used to explore possible causes of heterogeneity among study results (e.g. subgroup analysis, meta-regression).	11
	13f	Describe any sensitivity analyses conducted to assess robustness of the synthesized results.	11
Reporting bias assessment	14	Describe any methods used to assess risk of bias due to missing results in a synthesis (arising from reporting biases).	(Supp. Table 2)
Certainty assessment	15	Describe any methods used to assess certainty (or confidence) in the body of evidence for an outcome.	3-11 (Supp. Table 2)
**RESULTS**			
Study selection	16a	Describe the results of the search and selection process, from the number of records identified in the search to the number of studies included in the review, ideally using a flow diagram.	3 (Figure 1)
	16b	Cite studies that might appear to meet the inclusion criteria, but which were excluded, and explain why they were excluded.	n/a
Study characteristics	17	Cite each included study and present its characteristics.	5-11 (Table 4)
Risk of bias in studies	18	Present assessments of risk of bias for each included study.	(Supp. Table 1)
Results of individual studies	19	For all outcomes, present, for each study: (a) summary statistics for each group (where appropriate) and (b) an effect estimate and its precision (e.g. confidence/credible interval), ideally using structured tables or plots.	Tables 5-7
Results of syntheses	20a	For each synthesis, briefly summarise the characteristics and risk of bias among contributing studies.	Supp. Table 2
	20b	Present results of all statistical syntheses conducted. If meta-analysis was done, present for each the summary estimate and its precision (e.g. confidence/credible interval) and measures of statistical heterogeneity. If comparing groups, describe the direction of the effect.	Table 8
	20c	Present results of all investigations of possible causes of heterogeneity among study results.	11-12
	20d	Present results of all sensitivity analyses conducted to assess the robustness of the synthesized results.	11-12
Reporting biases	21	Present assessments of risk of bias due to missing results (arising from reporting biases) for each synthesis assessed.	11-12
Certainty of evidence	22	Present assessments of certainty (or confidence) in the body of evidence for each outcome assessed.	11-12
**DISCUSSION**			
Discussion	23a	Provide a general interpretation of the results in the context of other evidence.	13

Section and Topic	Item #	Checklist item	Reported on page # (or table #)
	23b	Discuss any limitations of the evidence included in the review.	13
	23c	Discuss any limitations of the review processes used.	13
	23d	Discuss implications of the results for practice, policy, and future research.	13
**OTHER INFORMATION**			
Registration and protocol	24a	Provide registration information for the review, including register name and registration number, or state that the review was not registered.	2
	24b	Indicate where the review protocol can be accessed, or state that a protocol was not prepared.	2
	24c	Describe and explain any amendments to information provided at registration or in the protocol.	2
Support	25	Describe sources of financial or non-financial support for the review, and the role of the funders or sponsors in the review.	n/a
Competing interests	26	Declare any competing interests of review authors.	14
Availability of data, code and other materials	27	Report which of the following are publicly available and where they can be found: template data collection forms; data extracted from included studies; data used for all analyses; analytic code; any other materials used in the review.	n/a

From: Page MJ, McKenzie JE, Bossuyt PM, Boutron I, Hoffmann TC, Mulrow CD, et al. The PRISMA 2020 statement: an updated guideline for reporting systematic reviews. BMJ 2021;372:n71. doi: 10.1136/bmj.n71

For more information, visit: http://www.prisma-statement.org/

Supplementary table 3. Quality assessment of the 38 studies included into the systematic review evaluated by the checklist proposed by Fowkes et al. [13], indicating found flaws in the study design or reporting.

Reference	Study design appropriate to objectives	Study sample representative					Control group acceptable				Quality of measurements and outcome				Completeness				Distorting influences				
	Objective: Cause	*Source of sample*	*Sampling method*	*Sample size*	*Non respondents*	*Entry criteria/exclusion*	*Definition of controls*	*Source of controls*	*Matching/randomization*	*Comparable characteristics*	*Validity*	*Reproducibility*	*Blindness*	*Quality control*	*Compliance*	*Dropouts*	*Deaths*	*Missing data*	*Extraneous treatments*	*Contamination*	*Changes over time*	*Confounding factors*	*Distortion reduced by analysis*
Abudaowd et al., 2021 [14]	0	0	0	0	+	0	0	0	0	0	0	0	NA	0	NA	NA	NA	0	NA	NA	NA	+	0
Altinkaynak et al., 2006 [15]	0	0	0	0	+	0	0	0	0	0	0	0	NA	0	NA	NA	NA	0	NA	NA	NA	+	0
Amitay et al., 2016 [16]	0	0	0	0	+	0	0	0	0	0	0	0	NA	0	NA	NA	NA	0	NA	NA	NA	+	0
Ansell et al., 1996 [43]	0	0	0	0	NA	0	0	0	0	0	0	0	NA	0	NA	NA	NA	+	NA	NA	NA	+	0
Bener et al., 2001 [17]	0	0	0	0	+	0	0	0	0	0	0	0	NA	0	NA	NA	NA	0	NA	NA	NA	+	0
Bener et al., 2008 [18]	0	0	0	0	+	0	0	0	0	0	0	0	NA	0	NA	NA	NA	0	NA	NA	NA	+	0
Bonaventure et al., 2012 [19]	0	0	0	0	0	0	0	0	0	0	0	0	NA	0	NA	NA	NA	0	NA	NA	NA	+	0
Davis et al, 1988 [20]	0	0	0	0	0	0	0	0	0	0	0	0	NA	0	NA	NA	NA	0	NA	NA	NA	+	0
Diamantaras et al, 2013 [39]	0	0	0	0	0	0	0	0	0	0	0	0	NA	0	NA	NA	NA	0	NA	NA	NA	+	0
Ekelund et al., 1993 [44]	0	0	0	0	NA	0	0	0	0	0	0	+	NA	+	NA	NA	NA	0	NA	NA	NA	+	0
Fear et al., 2003 [45]	0	0	0	0	0	0	0	0	0	0	0	0	NA	0	NA	NA	NA	0	NA	NA	NA	+	0
Francis et al., 2014 [21]	0	0	0	0	0	0	0	0	0	0	0	0	NA	0	NA	NA	NA	0	NA	NA	NA	+	0
Gao et al., 2018 [22]	0	0	0	0	0	0	0	0	0	0	0	0	NA	0	NA	NA	NA	0	NA	NA	NA	+	0
Golding et al., 1992 [46]	0	0	0	0	0	0	0	0	0	0	0	0	NA	0	NA	NA	NA	0	NA	NA	NA	+	0
Greenop et al., 2015 [23]	0	0	0	0	0	0	0	0	0	0	0	0	NA	0	NA	NA	NA	0	NA	NA	NA	+	0
Hardell et al., 2001 [24]	0	0	0	0	0	0	0	0	0	0	0	0	NA	0	NA	NA	NA	0	NA	NA	NA	0	0
Infante-Rivard et al., 2000 [25]	0	0	0	0	0	0	0	0	0	0	0	0	NA	0	NA	NA	NA	0	NA	NA	NA	0	0
Kwan et al., 2004 [40]	0	0	0	0	0	0	0	0	0	0	+	0	NA	0	NA	NA	NA	0	NA	NA	NA	+	0
Kwan et al., 2005 [26]	0	0	0	0	0	0	0	0	0	0	+	0	NA	0	NA	NA	NA	0	NA	NA	NA	+	0
Lancashire et al., 2003 [27]	0	0	0	0	+	0	0	0	0	0	+	0	NA	0	NA	NA	NA	0	NA	NA	NA	+	0
Lingappa et al., 2018 [28]	0	0	0	0	+	0	0	0	0	0	+	0	NA	0	NA	NA	NA	0	NA	NA	NA	+	0
Liu et al., 2009 [41]	0	0	0	0	0	0	0	0	0	0	0	0	NA	0	NA	NA	NA	0	NA	NA	NA	+	0

Supplementary table 3. Quality assessment of the 38 studies included into the systematic review evaluated by the checklist proposed by Fowkes et al. [13], indicating found flaws in the study design or reporting.

Reference	Study design appropriate to objectives	Study sample representative					Control group acceptable				Quality of measurements and outcome				Completeness				Distorting influences				
	Objective: Cause	*Source of sample*	*Sampling method*	*Sample size*	*Non respondents*	*Entry criteria/exclusion*	*Definition of controls*	*Source of controls*	*Matching/randomization*	*Comparable characteristics*	*Validity*	*Reproducibility*	*Blindness*	*Quality control*	*Compliance*	*Dropouts*	*Deaths*	*Missing data*	*Extraneous treatments*	*Contamination*	*Changes over time*	*Confounding factors*	*Distortion reduced by analysis*
MacArthur et al., 2008 [29]	0	0	0	0	0	0	0	0	0	+	+	0	NA	0	NA	NA	NA	0	NA	NA	NA	+	0
McKinney et al, 1998 [47]	0	0	0	0	0	0	0	0	0	0	0	0	NA	0	NA	NA	NA	0	NA	NA	NA	+	0
Mohammadi et al., 2018 [30]	0	0	0	0	+	0	0	0	0	0	0	0	NA	0	NA	NA	NA	0	NA	NA	NA	++	0
Orsi et al., 2015 [31]	0	0	0	0	0	0	0	0	0	0	0	0	NA	0	NA	NA	NA	0	NA	NA	NA	+	0
Parker et al., 1998 [48]	0	0	0	0	NA	0	0	0	0	0	0	0	NA	0	NA	NA	NA	0	NA	NA	NA	+	0
Passmore et al., 1998 [49]	0	0	0	0	NA	0	0	0	0	0	0	0	NA	0	NA	NA	NA	0	NA	NA	NA	+	0
Perrilat et al., 2002 [32]	0	0	0	0	0	+	0	+	0	0	0	0	NA	0	NA	NA	NA	0	NA	NA	NA	+	0
Petridou et al., 1997 [33]	0	0	0	0	0	0	0	+	0	0	0	0	NA	0	NA	NA	NA	0	NA	NA	NA	+	0
Roman et al., 2002 [50]	0	0	0	0	NA	0	0	0	0	0	0	0	NA	0	NA	NA	NA	+	NA	NA	NA	+	0
Sarasua et al., 1993 [42]	0	0	0	++	0	0	0	0	0	0	0	0	NA	0	NA	NA	NA	0	NA	NA	NA	+	0
Saravia-Bartra et al, 2021 [34]	0	0	+	0	0	0	0	0	0	+	0	0	NA	0	NA	NA	NA	0	NA	NA	NA	+	0
Shu et al., 1995 [35]	0	0	0	0	0	0	0	0	0	+	+	0	NA	0	NA	NA	NA	0	NA	NA	NA	+	0
Shu et al., 1999 [36]	0	0	0	0	+	0	0	0	0	0	+	0	NA	0	NA	NA	NA	0	NA	NA	NA	+	0
U. K. Childhood Cancer Study Investigators, 2001 [37]	0	0	0	0	+	0	0	0	0	+	0	0	NA	0	NA	NA	NA	0	NA	NA	NA	0	0
Van Duijn et al., 1988 [38]	0	0	0	0	+	0	0	0	0	+	0	0	NA	0	NA	NA	NA	+	NA	NA	NA	0	0
von Kries et al., 1996 [51]	0	0	0	0	0	0	0	0	0	+	+	0	NA	0	NA	NA	NA	0	NA	NA	NA	+	0

Legend: 0=no flaw, +=minor flaw, ++=major flaw, NA= Not applicable.

Supplementary table 4. List of excluded articles, specifying the reason for exclusion

Author (s)	Journal	Cause of exclusion	Note/Extra information
Adzhimamudova, T. S., et al. (1987).	Gematol Transfuziol	Full text not available	
Alderson, M. (1980).	Adv Cancer Res	Full text not available	
Alexander, F. E. (1993).	Eur J Cancer	Study on infections	
Alexander, F. E., et al. (1997).	Br J Cancer	Excluded after quality assessment	The risk factor of interest was not evaluated
Alexander, F. E., et al. (1998).	Br J Cancer	Study on infections	
Alexander, F. E., et al. (1998).	Br J Cancer	Excluded after quality assessment	The risk factor of interest was not evaluated
Altieri, A., et al. (2006).	Cancer Epidemiol Biomarkers Prev 15(7): 1281-1286.	Excluded after quality assessment	The risk factor of interest was not evaluated
Ambs, E. (1966).	Med Welt	Full text not available	
Amitay, E. and L. Keinan-Boker (2014).	Harefuah	Publication type	
Andersen, M. (1993).	Sygeplejersken	Study on radiation	
Aoki, K. and H. Shimizu (1977).	Natl Cancer Inst Monogr	Full text not available	
Aquino, V. M. (2002).	Curr Probl Pediatr Adolesc Health Care	Full text not available	
Austin, D. F., et al. (1975).	Am J Epidemiol 101(1): 77-83.	Excluded after quality assessment	The risk factor of interest was not evaluated
Austin, D. F., et al. (1981).	Front Radiat Ther Oncol	Full text not available	
Auvinen, A. (1994).	Duodecim	Study on radiation	
Badaloni, C., et al. (2013).	Occup Environ Med	Study on environmental exposure	
Bailey, H. D., et al. (2011).	Paediatr Perinat Epidemiol	Study on environmental exposure	
Bailey, H. D., et al. (2014).	Cancer Causes Control	Study on environmental exposure	
Bartley, K., et al. (2010).	Int J Epidemiol	Study on radiation	
Ben-Bassat, I. and R. P. Gale (1984).	Leuk Res	Full text not available	
Beral, V. (1990).	Am J Epidemiol	Study on radiation	
Birch, J. M., et al. (2000).	Br J Cancer	Study on infections	
Bithell, J. F., et al. (1994).	BMJ	Study on radiation	
Bithell, J. F., et al. (2013).	Br J Cancer	Study on radiation	

Author (s)	Journal	Cause of exclusion	Note/Extra information
Boice, J. D., Jr. and R. W. Miller (1999).	Teratology	Study on radiation	
Bonilla, M., et al. (2000).	J Pediatr Hematol Oncol	Excluded after quality assessment	The risk factor of interest was not evaluated
Booth, B. J., et al. (2015).	Environ Health	Study on environmental exposure	
Bosco, J., et al. (1985).	Leuk Res	Full text not available	
Bouges, S., et al. (1999).	Rev Epidemiol Sante Publique	Study on radiation	
Boutou, O., et al. (2002).	Br J Cancer	Study on radiation	
Bross, I. D. and N. Natarajan (1972).	N Engl J Med	Study on radiation	
Bross, I. D. and R. Gibson (1970).	J Med	Full text not available	
Brosselin, P., et al. (2009).	Occup Environ Med	Study on environmental exposure	
Brown, R. C., et al. (2007).	Int J Epidemiol	Publication type	
Browning, D. and S. Gross (1968).	Am J Clin Child	Full text not available	
Burch, P. R., et al. (1970).	Lancet	Study on radiation	
Busby, C. and M. S. Cato (1998).	BMJ	Study on radiation	
Bussel, A., et al. (1978).	Nouv Rev Fr Hematol	Study on infections	
Cardwell, C. R., et al. (2008).	Br J Cancer	Study on infections	
Carlos-Wallace, F. M., et al. (2016).	Am J Epidemiol	Study on environmental exposure	
Caughey, R. W. and K. B. Michels (2009).	Int J Cancer	Publication type	
Chang, J. S., et al. (2009).	Blood Cells Mol Dis	Publication type	
Chang, J. S., et al. (2012).	Am J Epidemiol 176(11): 970-978.	Excluded after quality assessment	The risk factor of interest was not evaluated
Cheng, J., et al. (2014).	Am J Obstet Gynecol	Publication type	
Chokkalingam, A. P., et al. (2013).	Cancer Epidemiol Biomarkers Prev 22(6): 1088-1094.	Excluded after quality assessment	The risk factor of interest was not evaluated
Choudhry, V. P., et al. (1988).	Indian Pediatr	Full text not available	
Clavell, L. A. (1983).	Bol Asoc Med P R	Full text not available	
Cnattingius, S., et al. (1995).	J Natl Cancer Inst 87(12): 908-914.	Excluded after quality assessment	The risk factor of interest was not evaluated
Cocco, P., et al. (1996).	Leukemia	Full text not available	

Author (s)	Journal	Cause of exclusion	Note/Extra information
Colby-Graham, M. F. and C. Chordas (2003).	J Pediatr Nurs	Publication type	
Conard, R. A. (1975).	JAMA	Study on radiation	
Cook-Mozaffari, P., et al. (1989).	Lancet	Study on radiation	
Copeman, M. and L. DeSilva (1994).	Leukemia	Study on infections	
Costas, K., et al. (2002).	Sci Total Environ	Study on environmental exposure	
Couto, A. C., et al. (2015).	Eur J Cancer Prev 24(3): 245-252.	Excluded after quality assessment	The risk factor of interest was not evaluated
Craft, A. W. (1998).	Indian J Pediatr	Publication type	
Cronkite, E. P., et al. (1995).	Stem Cells	Study on radiation	
Crosignani, P., et al. (2004).	Int J Cancer	Study on environmental exposure	
Crump, C., et al. (2015).	Eur J Epidemiol 30(12): 1277-1285.	Excluded after quality assessment	The risk factor of interest was not evaluated
Darby, S. C. and E. Roman (1996).	Nature	Full text not available	
Darby, S. C., et al. (1992).	BMJ	Study on radiation	
Davies, S. M. and J. A. Ross (2003).	Med Pediatr Oncol	Publication type	
Davis, S., et al. (2006).	Int J Epidemiol	Study on radiation	
Deziel, N. C., et al. (2014).	Environ Res	Study on environmental exposure	
Dickinson, H. O. (2005).	BMJ	Publication type	
Dockerty, J. D. (2009).	Blood Cells Mol Dis	Study on infections	
Dockerty, J. D., et al. (1996).	Br J Cancer 73(9): 1141-1147.	Excluded after quality assessment	The risk factor of interest was not evaluated
Dockerty, J. D., et al. (1998).	Cancer Causes Control	Study on environmental exposure	
Doyle, P. and E. Roman (1993).	BMJ	Study on radiation	
Draper, G. J., et al. (1993).	BMJ	Study on radiation	
Draper, G. J., et al. (1997).	BMJ	Study on radiation	
Eatough, J. P. and D. L. Henshaw (1994).	Int J Epidemiol	Study on radiation	
Emanuel, P. D. (2007).	Curr Hematol Malig Rep	Publication type	
Ennis, J. and C. R. Muirhead (1985).	Lancet	Study on radiation	

Author (s)	Journal	Cause of exclusion	Note/Extra information
Evans, H. J. (1990).	Nature	Study on radiation	
Fairlie, I. (2010).	Int J Occup Environ Health	Study on radiation	
Fajardo-Gutierrez, A., et al. (1993).	Bol Med Hosp Infant Mex 50(4): 248-257.	Excluded after quality assessment	The risk factor of interest was not evaluated
Feliu, J., et al. (1988).	Cancer	Excluded after quality assessment	The risk factor of interest was not evaluated
Feychting, M., et al. (1995).	Eur J Cancer	Study on environmental exposure	
Feychting, M., et al. (2000).	Cancer Causes Control	Study on environmental exposure	
Finlay, J. L. and W. Borcherding (1988).	Leukemia	Excluded after quality assessment	The risk factor of interest was not evaluated
Foliart, D. E., et al. (2001).	Bioelectromagnetics	Study on environmental exposure	
Gardner, M. J. (1992).	J Natl Cancer Inst	Study on radiation	
Gefferth, K. (1968).	Strahlentherapie	Study on radiation	
Gilham, C., et al. (2005).	BMJ 330(7503): 1294.	Excluded after quality assessment	The risk factor of interest was not evaluated
Glass, D. C., et al. (2012).	Occup Environ Med	Study on environmental exposure	
Gradel, K. O. and L. Kaerlev (2015).	Pediatr Blood Cancer 62(7): 1155-1161.	Excluded after quality assessment	The risk factor of interest was not evaluated
Greaves, M. (2002).	BMJ	Publication type	
Greaves, M. (2006).	Discov Med	Publication type	
Greaves, M. and P. A. Buffler (2009).	Br J Cancer	Study on infections	
Greaves, M. F. and F. E. Alexander (1993).	Leukemia	Study on infections	
Green, L. M., et al. (1999).	Cancer Causes Control	Study on environmental exposure	
Green, L. M., et al. (1999).	Int J Cancer	Study on environmental exposure	
Groves, F. D., et al. (2001).	Br J Cancer	Study on infections	
Gustafsson, M. and W. Mortensson (1983).	Acta Radiol Diagn	Study on radiation	
Hakulinen, T., et al. (1973).	Br Med J	Study on infections	

Author (s)	Journal	Cause of exclusion	Note/Extra information
Hansen, M. M. (1973).	Scand J Haematol	Full text not available	
Hashizume, T. (1981).	Rinsho Hoshasen	Study on radiation	
Heck, J. E., et al. (2014).	Int J Hyg Environ Health	Study on environmental exposure	
Heegaard, E. D., et al. (1999).	Pediatr Hematol Oncol	Study on infections	
Herrmann, T. (1980).	Radiobiol Radiother (Berl	Study on radiation	
Hewitt, D., et al. (1966).	Cancer	Full text not available	
Hjalgrim, L. L., et al. (2003).	Am J Epidemiol	Publication type	
Hoffmann, W. (2002).	Eur J Public Health	Study on radiation	
Hoffmann, W., et al. (1993).	Environ Health Perspect	Study on radiation	
Hood, E. (2003).	Environ Health Perspect	Study on environmental exposure	
Hopton, P. A., et al. (1985).	Lancet	Study on radiation	
Houot, J., et al. (2015).	Am J Epidemiol	Study on environmental exposure	
Hung, I. J., et al. (1982).	Taiwan Yi Xue Hui Za Zhi	Full text not available	
Hurtig, A. K. and M. San Sebastian (2004).	Int J Occup Environ Health	Study on environmental exposure	
Huth, E. (1957).	Kinderarztl Prax	Study on infections	
Ibrahem, W. N., et al. (2014).	J Pak Med Assoc	Study on infections	
Inaba, H., et al. (2013).	Lancet	Publication type	
Infante-Rivard, C. and D. Sinnett (1999).	Lancet	Study on environmental exposure	
Infante-Rivard, C. and J. E. Deadman (2003).	Epidemiology	Study on environmental exposure	
Infante-Rivard, C., et al. (2001).	Epidemiology	Study on environmental exposure	
Infante-Rivard, C., et al. (2005).	Environ Health Perspect	Study on environmental exposure	
Isa, A., et al. (2004).	Pediatr Blood Cancer	Study on infections	
Ivanov, E. P., et al. (1993).	Nature	Study on radiation	
Jirik, V., et al. (2012).	Biomed Environ Sci	Study on environmental exposure	
Jurek, A. M., et al. (2009).	J Epidemiol Community Health	Publication type	

Author (s)	Journal	Cause of exclusion	Note/Extra information
Kaatsch, P., et al. (1998).	Cancer Causes Control	Study on radiation	
Kaatsch, P., et al. (2008).	Int J Cancer	Study on radiation	
Kamper-Jorgensen, M., et al. (2008).	Leukemia 22(1): 189-193.	Excluded after quality assessment	The risk factor of interest was not evaluated
Kappy, M. S. (1984).	Am J Clin Child	Study on radiation	
Kato, H. (1977).	Natl Cancer Inst Monogr	Study on radiation	
Kaune, W. T., et al. (2002).	Bioelectromagnetics	Study on environmental exposure	
Keenan, P., et al. (1980).	Ir Med J	Full text not available	
Keith, L. and E. Brown (1971).	Acta Genet Med Gemellol	Excluded after quality assessment	The risk factor of interest was not evaluated
Kendall, G., et al. (2011).	Leuk Res	Study on radiation	
Kerr, J. R., et al. (2003).	J Clin Pathol	Study on infections	
Khamsi, R. (2005).	Nature	Study on infections	
Kheifets, L., et al. (2015).	J Expo Sci Environ Epidemiol	Study on environmental exposure	
Kinlen, L. (1988).	Lancet	Study on infections	
Kinlen, L. J. (1993).	BMJ	Study on radiation	
Kinlen, L. J. (1998).	Cancer Causes Control	Study on infections	
Kinlen, L. J. (2015).	BMJ Open	Study on environmental exposure	
Kinlen, L. J. and A. Balkwill (2001).	Lancet	Study on infections	
Kinlen, L. J., et al. (1990).	Lancet	Study on infections	
Kinlen, L. J., et al. (1993).	BMJ	Study on radiation	
Kinlen, L., et al. (2002).	Br J Cancer	Study on environmental exposure	
Kneale, G. W. and A. M. Stewart (1976).	J Natl Cancer Inst	Study on radiation	
Knox, E. G., et al. (1980).	Br J Cancer	Study on infections	
Knusli, C. and M. Walter (2013).	Ther Umsch	Study on radiation	
Kumar, A., et al. (2014).	Asian Pac J Cancer Prev 15(2): 781-784.	Excluded after quality assessment	The risk factor of interest was not evaluated
Kurtzman, G. J., et al. (1988).	Lancet	Study on infections	

Author (s)	Journal	Cause of exclusion	Note/Extra information
Langholz, B., et al. (2002).	Ann Epidemiol	Study on environmental exposure	
Latino-Martel, P., et al. (2010).	Cancer Epidemiol Biomarkers Prev	Publication type	
Lawrence, G. A. (1979).	Hosp Pract	Study on radiation	
Lehtinen, M., et al. (2003).	Am J Epidemiol	Study on infections	
Lehtinen, M., et al. (2005).	Am J Epidemiol	Study on infections	
Lightfoot, T. (2005).	Bioelectromagnetics Suppl 7: S5-S	Publication type	
Lightfoot, T. J. and E. Roman (2004).	Toxicol Appl Pharmacol	Publication type	
Linet, M. S., et al. (1997).	N Engl J Med	Study on environmental exposure	
Little, M. P., et al. (1995).	Health Phys	Study on radiation	
Lombardi, C., et al. (2013).	Cancer Epidemiol Biomarkers Prev	Study on radiation	
London, S. J., et al. (1991).	Am J Epidemiol	Study on environmental exposure	
Lubin, J. H., et al. (1998).	J Natl Cancer Inst	Study on radiation	
Lupatsch, J. E., et al. (2015).	Eur J Epidemiol 30(12): 1287-1298.	Excluded after quality assessment	The risk factor of interest was not evaluated
Ma, X., et al. (2002).	Environ Health Perspect	Study on environmental exposure	
Ma, X., et al. (2009).	Blood Cells Mol Dis	Publication type	
Madon, E., et al. (1994).	Minerva Pediatr	Full text not available	
McBride, M. L., et al. (1999).	Am J Epidemiol	Study on environmental exposure	
McKenzie, D. R., et al. (1998).	Aust N Z J Public Health	Study on radiation	
McKinney, P. A., et al. (1987).	Arch Dis Child 62(3): 279-287.	Excluded after quality assessment	The risk factor of interest was not evaluated
McKinney, P. A., et al. (1995).	Health Bull	Study on environmental exposure	
McKinney, P. A., et al. (1999).	Br J Cancer 80(11): 1844-1851.	Excluded after quality assessment	The risk factor of interest was not evaluated
McKinney, P. A., et al. (2003).	Occup Environ Med	Study on environmental exposure	
McLaughlin, J. R., et al. (1993).	BMJ	Study on radiation	
McNally, R. J. and T. O. Eden (2004).	Br J Haematol	Study on infections	

Author (s)	Journal	Cause of exclusion	Note/Extra information
Meinert, R., et al. (1999).	Cancer Epidemiol Biomarkers Prev	Study on radiation	
Menegaux, F., et al. (2006).	Occup Environ Med	Study on environmental exposure	
Merzenich, H., et al. (2008).	Am J Epidemiol	Study on environmental exposure	
Metayer, C., et al. (2013).	J Expo Sci Environ Epidemiol	Study on environmental exposure	
Metayer, C., et al. (2013).	Cancer Epidemiol	Publication type	
Miligi, L., et al. (2013).	Occup Environ Med	Study on environmental exposure	
Miller, D., et al. (1993).	Can J Public Health	Study on radiation	
Miller, G., et al. (1972).	J Pediatr	Study on infections	
Miller, R. W. (1995).	Environ Health Perspect	Study on radiation	
Milne, E., et al. (2006).	Int J Cancer	Study on environmental exposure	
Milne, E., et al. (2015).	Cancer Epidemiol Biomarkers Prev 24(1): 48-56.	Excluded after quality assessment	The risk factor of interest was not evaluated
Mole, R. H. (1986).	Br Med J	Study on radiation	
Mole, R. H. (1991).	Leukemia	Study on radiation	
Monge, P., et al. (2004).	Int J Occup Environ Health	Study on environmental exposure	
Monge, P., et al. (2005).	Ann Occup Hyg	Study on environmental exposure	
Muirhead, C. R. (1991).	Soz Praventivmed	Study on radiation	
Murray, R., et al. (1959).	N Engl J Med	Study on radiation	
Naumburg, E. (2002).	Lakartidningen 99(24): 2745-2747.	Excluded after quality assessment	The risk factor of interest was not evaluated
Naumburg, E., et al. (2000).	BMJ	Study on radiation	
Naumburg, E., et al. (2002).	Med Pediatr Oncol	Study on infections	
Neglia, J. P., et al. (2000).	Br J Cancer	Study on infections	
Noshchenko, A. G., et al. (2002).	Int J Cancer	Study on radiation	
Noshchenko, A. G., et al. (2010).	Int J Cancer	Study on radiation	
Nussbaum, R. H. and W. Kohnlein (1996).	Environ Health Perspect	Study on radiation	

Author (s)	Journal	Cause of exclusion	Note/Extra information
Nyari, T. A., et al. (2006).	J Perinat Med	Publication type	
Ogris, E. (1997).	Acta Med Austriaca	Study on radiation	
Ortega, J. J., et al. (1984).	Sangre	Full text not available	
Pearce, M. S., et al. (2012).	Lancet	Study on radiation	
Pedersen, C., et al. (2014).	PLoS One	Study on environmental exposure	
Perrillat, F., et al. (2002).	Br J Cancer	Study on infections	
Petridou, E. (2001).	Epidemiology	Study on environmental exposure	
Petridou, E. and N. Dessypris (2000).	Epidemiology	Study on environmental exposure	
Petridou, E., et al. (1993).	BMJ	Study on infections	
Petridou, E., et al. (1996).	Nature	Study on radiation	
Petridou, E., et al. (1997).	Int J Cancer	Study on environmental exposure	
Petridou, E., et al. (1997).	Cancer Causes Control	Excluded after quality assessment	The risk factor of interest was not evaluated
Petridou, E., et al. (2001).	Cancer Causes Control	Study on infections	
Pieters, R., et al. (1987).	Cancer	Study on environmental exposure	
Poole, C., et al. (2006).	Int J Epidemiol	Publication type	
Pui, C. H., et al. (2012).	Blood	Publication type	
Pui, C. H., et al. (2015).	J Clin Oncol	Publication type	
Rechavi, G., et al. (1992).	Acta Haematol	Study on infections	
Reeves, J. D., et al. (1981).	Lancet	Study on environmental exposure	
Repacholi, M. H. (2003).	Radiat Prot Dosimetry	Study on radiation	
Reynolds, P., et al. (2003).	Environ Health Perspect	Study on environmental exposure	
Roff, S. R. (2004).	Pediatrics	Publication type	
Rokicka-Milewska, R., et al. (1992).	Acta Haematol Pol	Study on infections	
Roman, E., et al. (1987).	Br Med J	Study on radiation	
Roman, E., et al. (1993).	BMJ 306(6878): 615-621.	Excluded after quality assessment	The risk factor of interest was not evaluated

Author (s)	Journal	Cause of exclusion	Note/Extra information
Roman, E., et al. (1994).	J Epidemiol Community Health 48(6): 601-602.	Excluded after quality assessment	The risk factor of interest was not evaluated
Roman, E., et al. (1997).	Br J Cancer 76(3): 406-415.	Excluded after quality assessment	The risk factor of interest was not evaluated
Roman, E., et al. (2009).	Blood Cells Mol Dis	Study on infections	
Rosenbaum, P. F., et al. (2005).	Paediatr Perinat Epidemiol	Study on infections	
Ross, J. A., et al. (1997).	Ann Epidemiol 7(3): 172-179.	Excluded after quality assessment	The risk factor of interest was not evaluated
Rossig, C. and H. Juergens (2008).	Radiat Prot Dosimetry	Publication type	
Rowley, J. D. (1998).	Nat Med	Publication type	
Ruckart, P. Z., et al. (2013).	Environ Health	Study on environmental exposure	
Russ, A. (2007).	Environ Health Perspect	Study on radiation	
Sali, D., et al. (1996).	Int J Cancer	Study on radiation	
Sasco, A. J. and H. Vainio (1999).	Hum Exp Toxicol	Publication type	
Schull, W. J. (1983).	Adv Space Res	Study on radiation	
Schuz, J., et al. (2000).	Cancer Epidemiol Biomarkers Prev	Study on environmental exposure	
Schuz, J., et al. (2001).	Int J Cancer	Study on environmental exposure	
Schuz, J., et al. (2008).	Am J Epidemiol	Study on radiation	
Sergentanis, T. N., et al. (2015).	Eur J Epidemiol 30(12): 1229-1261.	Excluded after quality assessment	The risk factor of interest was not evaluated
Sermage-Faure, C., et al. (2012).	Int J Cancer	Study on radiation	
Shu, X. O., et al. (1994).	Br J Cancer	Study on radiation	
Shu, X. O., et al. (1999).	Cancer Epidemiol Biomarkers Prev	Study on environmental exposure	
Shu, X. O., et al. (2002).	Cancer Epidemiol Biomarkers Prev	Study on radiation	
Simpson, J., et al. (2007).	Eur J Cancer	Study on infections	
Sipowicz, M. A. and T. Laudanski (2002).	Ginekol Pol	Publication type	
Skinner, J., et al. (2002).	Br J Cancer	Study on environmental exposure	
Smith, M. A., et al. (1997).	J Natl Cancer Inst	Publication type	

Author (s)	Journal	Cause of exclusion	Note/Extra information
Smith, M. A., et al. (1998).	Cancer Causes Control	Study on infections	
Smith, P. G. (1991).	BMJ	Publication type	
Soderberg, K. C., et al. (2002).	Epidemiology	Study on environmental exposure	
Sohrabi, M. R., et al. (2010).	Asian Pac J Cancer Prev	Study on environmental exposure	
Sorensen, H. T., et al. (2001).	JAMA	Excluded after quality assessment	The risk factor of interest was not evaluated
Spector, L. G., et al. (2007).	Cancer Epidemiol Biomarkers Prev 16(1): 128-134.	Excluded after quality assessment	The risk factor of interest was not evaluated
Speer, S. A., et al. (2002).	J Environ Health 64(7): 9-16; quiz 35-16.	Excluded after quality assessment	The risk factor of interest was not evaluated
Spix, C., et al. (2009).	Klin Padiatr 221(6): 362-368.	Excluded after quality assessment	The risk factor of interest was not evaluated
Spycher, B. D., et al. (2011).	Int J Epidemiol	Study on radiation	
Stewart, A. (1999).	Med Confl Surviv	Study on radiation	
Stewart, A. (2001).	Int J Epidemiol	Publication type	
Stjernfeldt, M., et al. (1986).	Lancet	Publication type	
Stjernfeldt, M., et al. (1992).	Cancer Detect Prev	Study on radiation	
Sung, T. I., et al. (2008).	Reprod Toxicol	Study on environmental exposure	
Tabrizi, M. M. and S. A. Hosseini (2015).	Asian Pac J Cancer Prev	Study on environmental exposure	
Tredaniel, J., et al. (1994).	Paediatr Perinat Epidemiol	Publication type	
Turner, M. C., et al. (2011).	Cien Saude Colet	Study on environmental exposure	
Valberg, P. A. (1996).	Int Arch Occup Environ Health	Study on environmental exposure	
van Steensel-Moll, H. A., et al. (1986).	Am J Epidemiol	Study on infections	
Vasconcelos, G. M., et al. (2011).	Epigenetics	Study on infections	
Vasilatou-Kosmidis, H. (2003).	Med Pediatr Oncol	Publication type	
Viel, J. F. and S. T. Richardson (1990).	BMJ	Study on radiation	
Wakeford, R. (1995).	Environ Health Perspect	Study on radiation	

**Author (s)**	**Journal**	**Cause of exclusion**	**Note/Extra information**
Wakeford, R. (2009).	Radiat Environ Biophys	Study on radiation	
Wakeford, R. (2014).	Radiat Environ Biophys	Study on radiation	
Wakeford, R. and M. P. Little (2003).	Int J Radiat Biol	Study on radiation	
Wakeford, R., et al. (2010).	Radiat Environ Biophys	Study on radiation	
Ward, M. H., et al. (2009).	Environ Health Perspect	Study on environmental exposure	
Webster, E. W. (1981).	Am J Roentgenol	Study on radiation	
Wen, W. Q., et al. (2000).	Am J Epidemiol	Study on environmental exposure	
Wen, W., et al. (2000).	Cancer Causes Control 11(4): 303-307.	Excluded after quality assessment	The risk factor of interest was not evaluated
Weng, H. H., et al. (2008).	Inhal Toxicol	Study on environmental exposure	
Whitehead, T. P., et al. (2015).	J Expo Sci Environ Epidemiol	Study on environmental exposure	
Whitehead, T., et al. (2009).	Am J Epidemiol	Excluded after quality assessment	The risk factor of interest was not evaluated
Wolff, S. P. (1991).	Nature	Full text not available	
Wood, D. J. and G. Corbitt (1985).	J Infect Dis	Study on infections	
Wunsch-Filho, V., et al. (2011).	Cancer Epidemiol	Study on environmental exposure	
Yasmeen, N. and S. Ashraf (2009).	J Pak Med Assoc	Publication type	
Yeazel, M. W., et al. (1995).	Cancer 75(7): 1718-1727.	Excluded after quality assessment	The risk factor of interest was not evaluated
Yu, C. L., et al. (2006).	Am J Epidemiol	Study on environmental exposure	
Zack, M., et al. (1991).	Cancer Res 51(14): 3696-3701.	Excluded after quality assessment	The risk factor of interest was not evaluated
Zahm, S. H. and M. H. Ward (1998).	Environ Health Perspect	Study on environmental exposure	
Zou, G. and X. Sha (2014).	Zhonghua Er Ke Za	Publication type	
Stjernfeldt et al., 1986	Lancet	Excluded after quality assessment	Data couldn't be extracted
Robison et al., 1989	Env Health Perspectives	Excluded after quality assessment	Data couldn't be extracted

Author (s)	Journal	Cause of exclusion	Note/Extra information
Schwartzbaum et al., 1991	Med and Pediatr Oncol	Excluded after quality assessment	Data couldn't be extracted
Olsen et al, 1994	BMJ	Excluded after quality assessment	Data couldn't be extracted
Klebanoff et al., 1996	Am J Epidemiol	Excluded after quality assessment	Data couldn't be extracted
(2000).	Br J Cancer	Study on environmental exposure	
Johnson et al., 2009	Epidemiology	Excluded after quality assessment	Data couldn't be extracted
Kennedy et al., 2015	J Pediatr Hematol Oncol	Excluded after quality assessment	Data couldn't be extracted
Kucukcongar et al., 2015	Pediatr Hematol Oncol	Excluded after quality assessment	Data couldn't be extracted
Yan et al., 2016	Pediatric Blood and Cancer	Publication type	
Dessypris et al., 2017	Int J of Cancer Epi	Publication type	
Karalexi et al., 2019	Cancer epidemiology	Publication type	
Ayub et al., 2020	J Pak Med Assoc	Excluded after quality assessment	Restricted to a center, limited number, lack of adjusted analysis

Supplementary table 5. Association between breastfeeding and the risk of acute leukemia in children

Reference	Type of Leukemia	Tested duration	Reference duration	OR/HR	95% CI	Adjustments
Abudaowd et al., 2021	Acute leukemia	Any	None	1.79	0.69-4.69	Univariate analysis
Altinkaynak et al, 2006	ALL	0-6 months	>6 months	2.44	1.47-5.10*	Gestational age, birth order, birth weight, maternal age and smoking during pregnancy.
	AML	0-6 months	>6 months	6.67	1.32-33.69*	
Amitay et al., 2016	Acute leukemia	≤6 months	> 6 month	0.53	0.33-0.85*	Paternal smoking for at least 3 mo; iron supplement; Socioeconomic status (based on a combination of family income and maternal education)
Bener et al., 2001	ALL	0-6 months	>6 months	2.47	1.17-5.25*	Age, sex and ethnicity
Bener et al., 2008	ALL	0-6 months	>6 months	2.90	1.60-5.30*	Sex and age.
Bonaventure et al., 2012	Acute leukemia	Any	None	0.70	0.60-0.90*	Gender and age.
Davis et al. 1988	ALL	≤6 months	> 6 month	1.95	0.86-4.40	Univariate analysis
Francis et al. 2014	ALL	Any	None	0.93	0.70-1.25	Age, sex, maternal race, and income
	AML	Any	None	0.83	0.43-1.58	
Gao et al. 2018	Acute leukemia	None	>13 months	8.023	4.620-13.930*	Family history of cancer, family history of neoplasm of the lymphatic/hematopoietic system, history of bedroom decoration, smoking during pregnancy, use of birth controls pills during pregnancy, abortion history, Down's syndrome, parents use of hairy dye
		1-3 months		1.037	0.636-1.691	
		4-6 months		0.751	0.479-1.178	
		7-9 months		0.498	0.318-0.780*	
		10-12 months		1.114	0.718-1.727	
Greenop et al., 2015	ALL	Any	No	0.52	0.32-0.86*	Child's age in months at diagnosis for cases and date of questionnaire return for controls, sex, and state, maternal education, maternal age at birth, birth order, proportion of optimum birth weight
		<3 months		0.49	0.28-0.86*	
		≥3 - <6 months		0.62	0.34-1.12	
		≥ 6 months		0.51	0.30-0.84*	
Hardell et al. 2001	Acute leukemia	1 - <6 months	<1 month	0.9	0.5-1.7	Maternal age, maternal smoking behaviour, gestational age, birth weight, order of birth
		≥ 6 months		0.9	0.5-1.7	
		≥ 1 month		0.9	0.5-1.6	
	ALL	1 - <6 months	<1 month	1.0	0.5-2.0	

Reference	Type of Leukemia	Tested duration	Reference duration	OR/HR	95% CI	Adjustments
		≥ 6 months		0.9	0.5-1.8	
		≥ 1 month		1.0	0.5-1.9	
	AML	1 - <6 months	<1 month	0.2	0.0-2.0	
		≥ 6 months		0.3	0.0-3.2	
		≥ 1 month		0.3	0.0-2.2	
Infante-Rivard et al., 2000	ALL	≤ 3 months	None	0.68	0.49-0.95*	Adjusted for maternal age and level of schooling
		> 3 months		0.67	0.47-0.94*	
Kwan et al., 2005	ALL	≤ 3 months	None	1.14	0.68-1.91	Adjusted for annual household income and maternal education
		4 - 6 months		0.84	0.48-1.47	
		7 – 12 months		0.88	0.51-1.53	
		≥ 13 months		1.08	0.61-1.92	
Lancashire et al. 2003	ALL	Any	None	0.99	0.82-1.20	Sex, age, social class, maternal age at birth, sibship position
		< 1 month		1.10	0.83-1.46	
		1-6 months		0.96	0.77-1.20	
		≥ 7 months		0.90	0.60-1.34	
	ANLL	Any	None	1.03	0.74-1.43	
		< 1 month		1.24	0.77-1.98	
		1-6 months		0.90	0.62-1.31	
		≥ 7 months		1.16	0.64-2.11	
Lingappa et al. 2018	Acute leukemia	Any	None	1	0.13-7.21	Univariate (but the controls were the siblings of the cases)
MacArthur et al. 2008	ALL	0-3 months	None	1.33	0.93-1.91	Maternal age, maternal education, annual household income, ethnicity, and number of residences since birth
		4-6 months		1.10	0.78-1.56	
		7-12 months		1.02	0.68-1.53	
Mohammadi et al. 2018	Acute leukemia	Any	None	0.34	0.10-1.10	Univariate analysis
		<1 month	20-24 months	2.87	0.91-8.96	
		1-6 months		0.92	0.44-1.93	
		7-12 months		1.28	0.59-2.77	
		13-19 months		1.14	0.61-2.13	
		≥24 months		1.51	0.59-3.87	

Reference	Type of Leukemia	Tested duration	Reference duration	OR/HR	95% CI	Adjustments
Orsi et al., 2015	Acute leukemia	Any	None	0.8	0.7-1.0*	Age and sex
	ALL	Any	None	0.8	0.7-1.0*	
	AML	Any	None	1.1	0.7-1.7	
Perrilat et al. 2002	ALL	Any	None	0.8	0.6-1.2	Sex, age, hospital, ethnicity, maternal education level and socio-professional category, gravidity, birthweight, order of birth
		< 3 months		1.0	0.6-1.6	
		3-5 months		1.3	0.8-2.4	
		6-11 months		0.5	0.2-1.1	
		≥12 months		0.5	0.1-2.5	
	ANLL	Any	None	0.8	0.6-1.2	
		< 3 months		1.6	0.5-5.2	
		3-5 months		0.8	0.1-3.8	
		6-11 months		0.5	0.1-3.2	
		≥12 months		1.1	0.1-17	
Petridou et al., 1997	Acute leukemia	Any	None	0.85	0.52-1.41	Gender, age and place of residence
Saravia Bartra et al., 2021	ALL	≥ 6 months	< 6 monts	0.56	0.35-0.90*	Gender, age, maternal age, maternal school level, region, traumatic event (physical or psicological event during prenancy)
Shu et al. 1995	Acute leukemia	Any	None	1.14	0.7-1.9	Maternal age at birth, birthweight, maternal working status and occupational exposure to chemicals during infancy
		1 - 6		1.20	0.6-2.3	
		> 6 months		1.11	0.6-1.9	
	ALL	Any	None	1.12	0.6-2.1	
		1 - 6		1.10	0.5-2.5	
		> 6 months		1.12	0.6-2.2	
	AML	Any	None	1.34	0.5-3.3	
		1 - 6		1.65	0.5-5.6	
		> 6 months		1.21	0.5-3.3	
Shu et al. 1999	ALL	Any	None	0.80	0.69-0.93*	Maternal education level, race, household income
		1-3 months		0.85	0.70-1.03	
		4-6 months		0.87	0.68-1.08	

Reference	Type of Leukemia	Tested duration	Reference duration	OR/HR	95% CI	Adjustments
		7-9 months		0.70	0.53-0.92*	
		10-12 months		0.61	0.46-0.80*	
		>12 months		0.85	0.66-1.11	
	AML	Any	None	0.77	0.57-1.03	
		1-3 months		1.12	0.73-1.72	
		4-6 months		0.81	0.54-1.23	
		7-9 months		0.48	0.28-0.82*	
		10-12 months		0.69	0.39-1.23	
		>12 months		0.58	0.31-1.08	
U. K. Childhood Cancer Study Investigators 2001	Acute leukemia	Any	None	0.89	0.80-1.00	Age at diagnosis, sex, region, order of birth, deprivation score
		< 1 month		0.96	0.81-1.14	
		1-6 months		0.88	0.77-1.02	
		≥ 7 months		0.85	0.73-1.00*	
	ALL	Any	None	0.91	0.81-1.04	
		< 1 month		0.96	0.82-1.17	
		1-6 months		0.90	0.77-1.04	
		≥ 7 months		0.89	0.75-1.05	
	AML	Any	None	0.78	0.58-1.05	
		< 1 month		0.82	0.53-1.26	
		1-6 months		0.85	0.60-1.20	
		≥ 7 months		0.65	0.43-1.00*	
Van Duijn et al., 1988	ALL	<6 months	None	1.15	0.80-1.67	Age, sex, birth order, social class. maternal education, and age, smoking, and alcohol use of mother during pregnancy
		≥ 6 months		0.83	0.48-1.43	

*=significant

Supplementary table 6. Association between early life diet and the risk of acute leukemia in children

Reference	Type of Leukemia	Food / Food group	Reference duration	OR/HR	95% CI	Adjustments
Amitay et al, 2016	Acute leukemia	Iron supplement	Yes	0.43	0.27-0.68*	Breastfeeding ≥6 mo; paternal smoking for at least 3 mo; and SES.
Diamantaras et al, 2013	ALL	Cereals and starchy roots	One quintile	1.17	0.92-1.49	Birth weight (500 g increment), birth order (one child more), maternal age (threeyear increment), maternal education (one level more) and tobacco smoking during pregnancy (yes vs. no) as well as for breastfeeding (yes vs. no)
		Sugars and syrups	One quintile	1.10	0.88-1.38	
		Pulses, nuts and seeds	One quintile	0.94	0.76-1.15	
		Vegetables	One quintile	1.09	0.90-1.33	
		Fruits	One quintile	1.05	0.88-1.25	
		Meats and meat products	One quintile	0.96	0.78-1.18	
		Fish and shellfish	One quintile	1.00	0.82-1.21	
		Milk and dairy products	One quintile	0.94	0.77-1.14	
		Added lipids	One quintile	1.31	1.04-1.64*	
Greenop et al., 2015	ALL	Cereals	>3.6 times/day	1.00	0.71-1.42	Adjusted for matching variables (child's age at infant feeding questionnaire return, sex, state of residence), maternal age, maternal education, birth order, proportion of optimum birth weight, child ever breast fed.
		Fruits	>2.6 times/day	1.14	0.85-1.65	
		Vegetables	>2.0 times/day	0.86	0.58-1.29	
		Dairy products	>3.7 times/day	1.13	0.80-1.61	
		Meat/fish/chicken/egg	>1.1 times/day	1.39	0.98-1.96	
Kwan et al., 2004	Acute leukemia	Hodtogs/launchmeats	Regular	1.61	0.72-3.58	Adjusted for birthweight (grams), duration of breastfeeding (months), maternal education (categorical), and annual household income (categorical). And Adjusted for the other foods/food groups (Model 2)
		Beef/hamburguer	Regular	1.56	0.79-3.06	
		Vegetables	Regular	0.66	0.31-1.40	
		Oranges/bananas	Regular	0.49	0.26-0.94*	
		Apples/grapes	Regular	0.94	0.47-1.85	
		Orange juice	Regular	0.54	0.31-0.94*	
		Fruit juice	Regular	1.42	0.77-2.62	
		Soda	Regular	1.31	0.53-3.27	
		Vitamins	Regular	0.70	0.42-1.18	
Liu et al., 2009	Acute leukemia	Cured meat/fish	Frequent	1.74	1.15-2.64*	Adjusted for age and sex
		Pickled vegetables	Frequent	1.10	0.62-1.93	
		Bean-curd food	Frequent	0.55	0.34-0.89*	
		Vegtables	Frequent	0.55	0.37-0.83*	
		Fruits	Frequent	0.86	0.58-1.26	

Reference	Type of Leukemia	Food / Food group	Reference duration	OR/HR	95% CI	Adjustments
		Tea	Yes	0.80	0.53-1.22	
Sarasua and Savitz, 1994	ALL	Ham, bacon, sausage	≥1 time/week	1.2	0.6-2.3	Adjusted for other types of meat (dichotomized), vitamin use, age at diagnosis, and *per capita* income
		Hot dogs	≥1 time/week	1.3	0.5-3.2	
		Hamburguers	≥1 time/week	2.0	0.9-4.6	
		Lunch meats	≥1 time/week	1.1	0.5-2.3	
		Charcoal broiled foods	Any	1.0	0.5-2.1	

*=significant

Supplementary table 7. Association between neonatal vitamin K administration and risk of acute leukemia in children

Reference	Type of Leukemia	Tested (vs none)	OR/RR	95% CI	Adjustments
Ansell et al. 1996	Acute leukemia	Intramuscular	1.30	0.70-2.30	Delivery type and admission to a special care nursery
	ALL		1.10	0.60-2.00	
Ekelund et al., 1993	Acute leukemia	Intramuscular	0.90	0.70-1.16	Year of birth.
Fear et al. 2003	Acute Leukemia$	Intramuscular	1		Sex, age at diagnosis (single years), region of residence
		Oral	1.16	0.88-1.42	
	ALL$	Intramuscular	1		
		Oral	1.10	0.86–1.41	
Golding et al., 1992	Acute leukemia	Intramuscular	2.65	1.34-5.24*	Hospital and year of birth
McKinney et al. 1998	Acute Leukemia	Intramuscular	1.23	0.77-1.97	Adjusted for deprivation category (affluent, standard, deprived) and type of delivery (normal, assisted, caesarean).
	ALL	Intramuscular	1.17	0.70-1.97	
	ALL$	Intramuscular	1.16	0.62-2.15	
Parker et al. 1998	ALL$	Intramuscular	1.79	1.02-3.15*	Opiate exposure and assisted delivery
Passmore et al., 1998	Acute leukemia	Intramuscular	1.53	0.82-2.85	Unadjusted
	ALL	Intramuscular	1.73	0.89-3.34	
Roman et al., 2002	Acute leukemia	Intramuscular	1.09	0.92-1.28	Adjusted for mode of delivery, admission to special care baby unit, low birth weight.
	ALL	Intramuscular	1.05	0.85-1.29	
von Kries et al., 1996	Acute leukemia	Intramuscular	0.98	0.64-1.50	Adjusted for type of region, social class, and prematurity. (local and state controls)

$ age at diagnosis ≥1 year. *= significant result

Meta-analysis of the association between breastfeeding and the risk of acute leukemia (lymphoblastic and myeloid)

Study or Subgroup	log[Odds Ratio]	SE	Weight	Odds Ratio IV, Random, 95% CI
Altinkaynak et al., 2006	0.892	0.375	16.6%	2.44 [1.17, 5.09]
Bener et al., 2008	1.0647	0.3034	19.1%	2.90 [1.60, 5.26]
Davis et al, 1988	0.6678	0.4177	15.3%	1.95 [0.86, 4.42]
Shu et al., 1999	-0.1508	0.0836	25.8%	0.86 [0.73, 1.01]
Van Duijn et al., 1988	0.1398	0.1852	23.2%	1.15 [0.80, 1.65]
**Total (95% CI)**			**100.0%**	**1.56 [0.96, 2.55]**

Heterogeneity: Tau² = 0.24; Chi² = 24.26, df = 4 (P < 0.0001); I² = 84%
Test for overall effect: Z = 1.79 (P = 0.07)

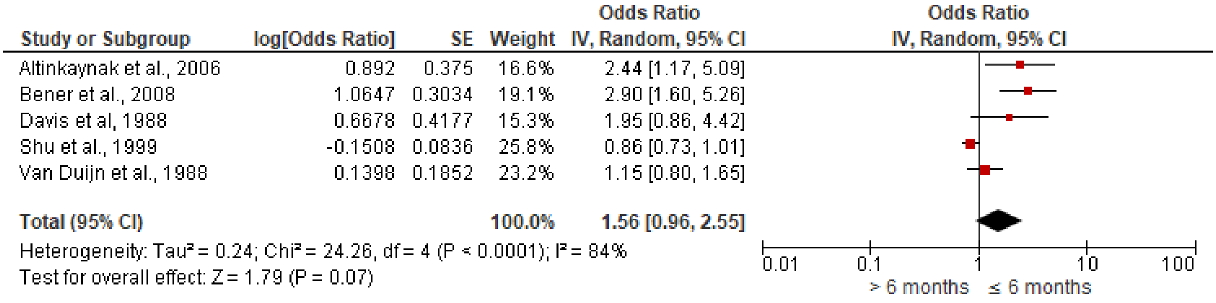

Supplementary figure 1: Random-effects model examining the association between breastfeeding (≤ 6 months) and risk of childhood ALL [15,18,20,36,38]. Note: the individual estimate (OR) from the studies is represented by the red box, and the black diamond represent the estimate of the meta-analysis.

Study or Subgroup	log[Odds Ratio]	SE	Weight	Odds Ratio IV, Random, 95% CI
Altinkaynak et al., 2006	1.8976	0.8265	41.4%	6.67 [1.32, 33.70]
Shu et al., 1999	-0.0513	0.1706	58.6%	0.95 [0.68, 1.33]
**Total (95% CI)**			**100.0%**	**2.13 [0.32, 13.97]**

Heterogeneity: Tau² = 1.54; Chi² = 5.33, df = 1 (P = 0.02); I² = 81%
Test for overall effect: Z = 0.79 (P = 0.43)

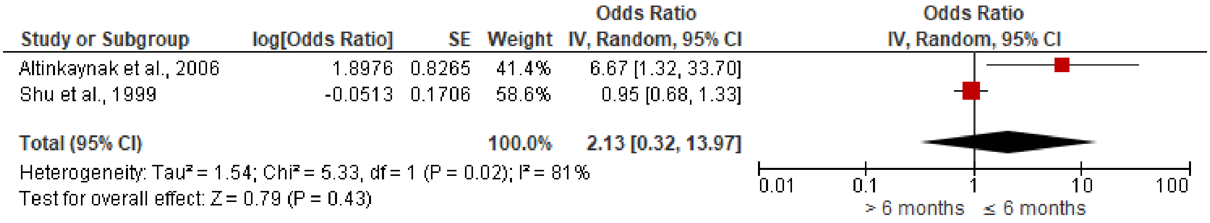

Supplementary figure 2: Random-effects model examining the association between breastfeeding (>6 versus ≤6 months) and risk of childhood AML [15,36]. Note: the individual estimate (OR) from the studies is represented by the red box, and the black diamond represent the estimate of the meta-analysis.

Study or Subgroup	log[Odds Ratio]	SE	Weight	Odds Ratio IV, Random, 95% CI
Francis et al., 2004	-0.0726	0.145	8.7%	0.93 [0.70, 1.24]
Greenop et al., 2015	-0.6539	0.2477	3.7%	0.52 [0.32, 0.85]
Kwan et al., 2005	-0.0101	0.2226	4.5%	0.99 [0.64, 1.53]
Lancashire et al., 2003	0.0392	0.097	14.0%	1.04 [0.86, 1.26]
Orsi et al., 2015	-0.2231	0.0681	18.8%	0.80 [0.70, 0.91]
Perrilat et al., 2002	-0.2231	0.1468	8.5%	0.80 [0.60, 1.07]
Saravia-Bartra et al, 2021	-0.5798	0.2398	4.0%	0.56 [0.35, 0.90]
Shu et al., 1999	-0.2231	0.0755	17.4%	0.80 [0.69, 0.93]
UKCCS, 2001	-0.0943	0.0594	20.4%	0.91 [0.81, 1.02]
**Total (95% CI)**			**100.0%**	**0.85 [0.76, 0.94]**

Heterogeneity: Tau² = 0.01; Chi² = 15.02, df = 8 (P = 0.06); I² = 47%
Test for overall effect: Z = 3.26 (P = 0.001)

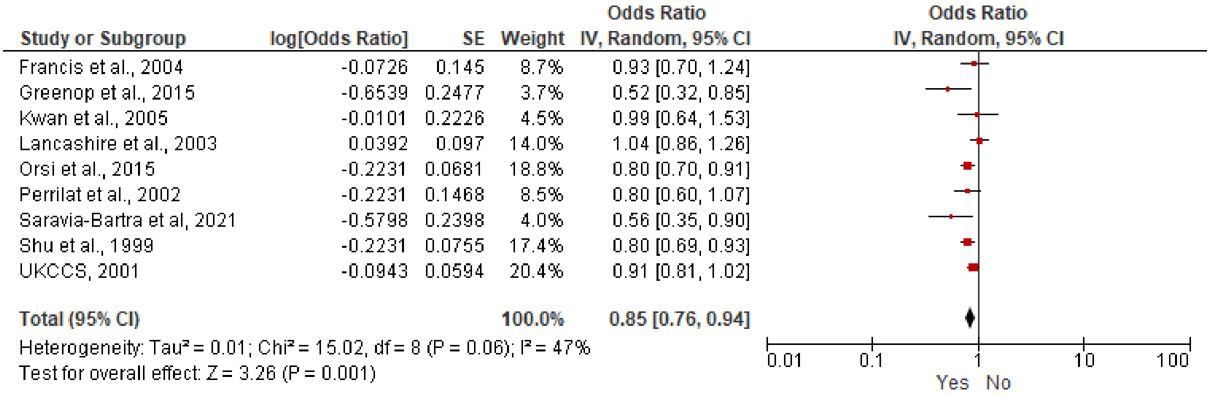

Supplementary figure 3: Random-effects model examining the association between breastfeeding (Yes versus No) and risk of childhood ALL [21,23,26,27,31,32,34,36,37]. Note: the individual estimate (OR) from the studies is represented by the red box, and the black diamond represent the estimate of the meta-analysis.

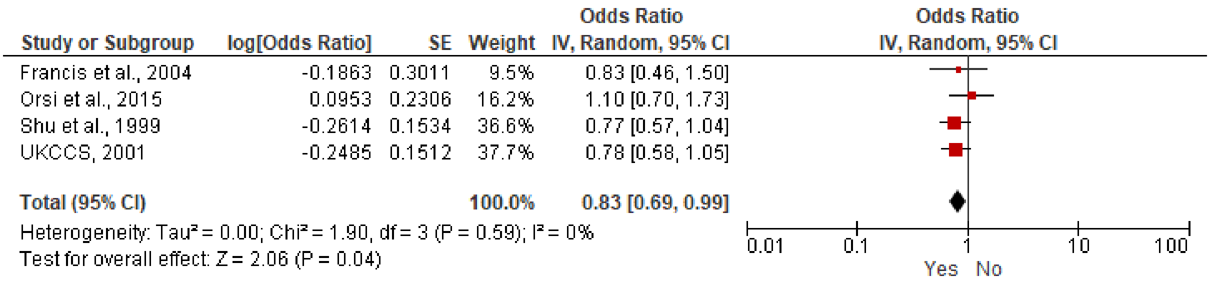

Supplementary figure 4: Random-effects model examining the association between breastfeeding (Yes versus No) and risk of childhood AML [21,31,36,37]. Note: the individual estimate (OR) from the studies is represented by the red box, and the black diamond represent the estimate of the meta-analysis.

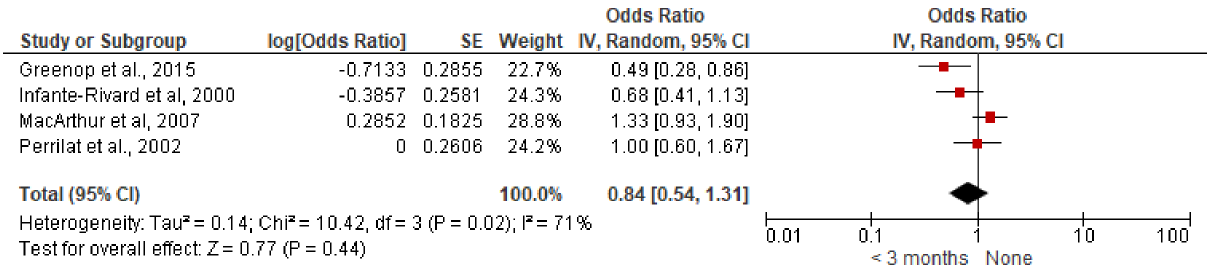

Supplementary figure 5: Random-effects model examining the association between breastfeeding (< 3 month) and risk of childhood ALL [23,25,29,32]. Note: the individual estimate (OR) from the studies is represented by the red box, and the black diamond represent the estimate of the meta-analysis.

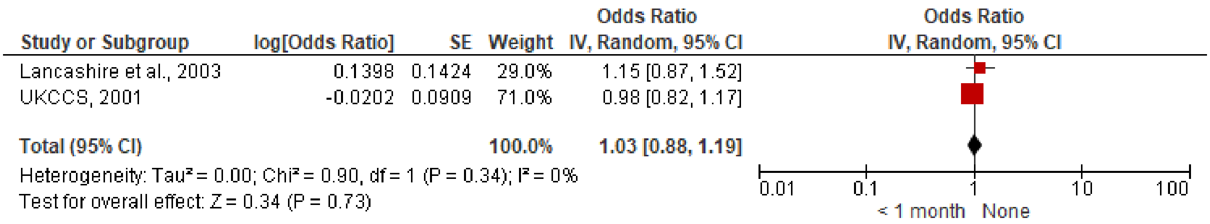

Supplementary figure 6: Random-effects model examining the association between breastfeeding (< 1 month) and risk of childhood ALL [27,37]. Note: the individual estimate (OR) from the studies is represented by the red box, and the black diamond represent the estimate of the meta-analysis.

Meta-analysis of the association between early life diet (first 2 years of life) and the risk of acute leukemia in children

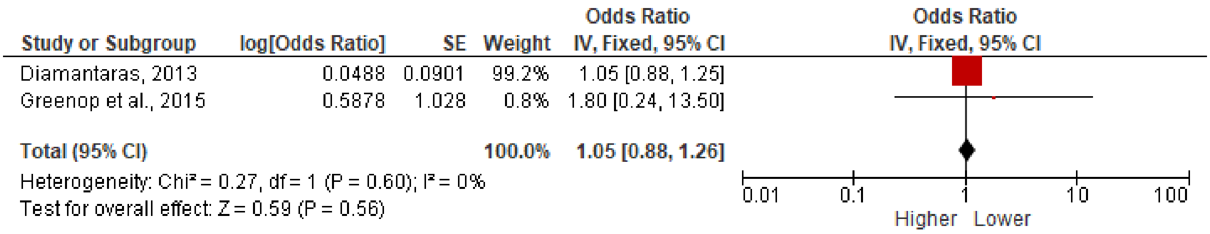

Supplementary figure 7. Random-effects model examining the association between the highest consumption of fruits (during the first 2 years of life) and risk of childhood ALL [23,39]. Note: the individual estimate (OR) from the studies is represented by the red box, and the black diamond represent the estimate of the meta-analysis.

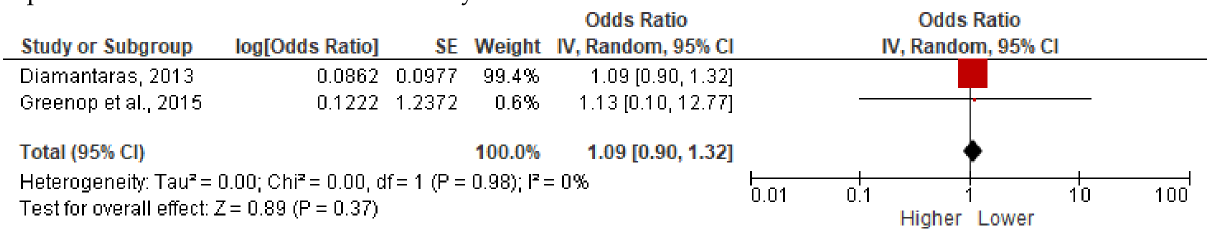

Supplementary figure 8. Random-effects model examining the association between the highest consumption of vegetables (during the first 2 years of life) and risk of childhood ALL [23,39]. Note: the individual estimate (OR) from the studies is represented by the red box, and the black diamond represent the estimate of the meta-analysis.

Meta-analysis of the association between vitamin k administration and childhood acute leukemia

Study or Subgroup	log[Odds Ratio]	SE	Weight	Odds Ratio IV, Random, 95% CI
Fear et al, 2003	-0.1138	0.0896	56.8%	0.89 [0.75, 1.06]
Roman et al, 2002	0.0488	0.1078	43.2%	1.05 [0.85, 1.30]
**Total (95% CI)**			**100.0%**	**0.96 [0.82, 1.12]**

Heterogeneity: Tau² = 0.00; Chi² = 1.35, df = 1 (P = 0.25); I² = 26%
Test for overall effect: Z = 0.54 (P = 0.59)

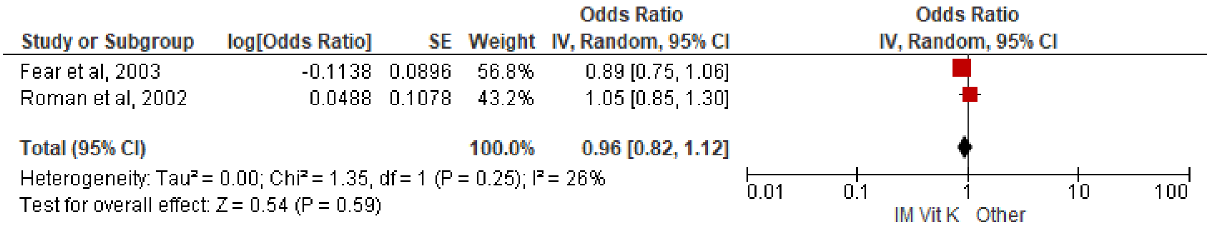

Supplementary figure 9. Random-effects model examining the association between intramuscular vitamin K given in neonatal period and risk of childhood ALL [45,50]. Note: the individual estimate (OR) from the studies is represented by the red box, and the black diamond represent the estimate of the meta-analysis.
